# Synthetic Opals or Versatile Nanotools—A One-Step Synthesis of Uniform Spherical Silica Particles

**DOI:** 10.3390/ijms241813693

**Published:** 2023-09-05

**Authors:** Magdalena Laskowska, Agnieszka Karczmarska, Mateusz Schabikowski, Michał Adamek, Alexey Maximenko, Katarzyna Pawlik, Oliwia Kowalska, Zbigniew Olejniczak, Łukasz Laskowski

**Affiliations:** 1 Institute of Nuclear Physics Polish Academy of Sciences, 31-342 Krakow, Poland; magdalena.laskowska@ifj.edu.pl (M.L.); agnieszka.karczmarska@ifj.edu.pl (A.K.); mateusz.schabikowski@ifj.edu.pl (M.S.); michal.adamek@ifj.edu.pl (M.A.); zbigniew.olejniczak@ifj.edu.pl (Z.O.); 2 SOLARIS National Synchrotron Radiation Centre, Jagiellonian University, 30-392 Krakow, Poland; alexey.maximenko@uj.edu.pl; 3 Faculty of Production Engineering and Materials Technology, Częstochowa University of Technology, 42-201 Częstochowa, Poland; katarzyna.pawlik@pcz.pl; 4 Jerzy Haber Institute of Catalysis and Surface Chemistry, Polish Academy of Sciences, 30-239 Krakow, Poland; oliwia.kowalska@ikifp.edu.pl

**Keywords:** artificial opals, nanoparticles, mesoparticles, silica, Stöber method

## Abstract

Synthetic opals, a composition of homogeneous silica spheres in the mesoscale size range, have attracted the attention of scientists due to their favorable chemical and physical properties. Their chemical inertness and stability, biocompatibility, homogeneity, elevated specific surface area, and ease of functionalization of their surfaces make them a versatile nanotool. In the present study, the Stöber process was used to investigate the effect of parameters, such as reagent concentration and synthesis temperature, on the resulting silica particle size and structure. The optimal conditions for successfully obtaining homogeneous particles in the mesoscale range with high reproducibility were investigated. Several synthesis procedures and their dependence on the reaction temperature were presented to allow the selection of the assumed diameter of silica spheres. The numerous samples obtained were examined for size, homogeneity, structure, and specific surface area. On the basis of specific surface area measurements and nuclear magnetic resonance studies, the internal hierarchical structure of the spherical silica was confirmed as consisting of a solid core and layers of secondary spheres covered by a solid shell. Structural studies (X-ray Spectroscopy, X-ray Absorption Near-Edge Structure, and nuclear magnetic resonance), together with infrared vibrational spectroscopy, showed no dependence of the structure of the obtained mesospheres on the concentration of reagents and the size of the obtained particles.

## 1. Introduction

Opals have attracted human attention for centuries because of their extraordinary optical properties. The uniqueness of these gemstones is due to a property called opalescence. It is characteristic of many nanostructured systems and is frequently observed, especially in biological systems like butterfly wing colors produced by chitin nanostructures [1,2].

In their natural and synthetic form, opals are solid colloidal crystals of self-assembled silica spheres [3], making them a key example of photonic crystals [4,5,6]. In this context, opalescence originates from the dependence of the spectral reflection or transmission band on the viewing angle [4]. The highly ordered three-dimensional structure of opals, which arises from the regular packing of spherical silica particles between 200 nm and 300 nm in diameter, reflects the visible light range of 400 nm to 700 nm [7,8].

The spatial arrangement of silica microspheres organized in cubic or hexagonal lattices causes iridescent properties attributed to optical interference. Still, their optical properties can also be considered in the context of the photonic band gap phenomenon [9]. Because silica opals with spherical particles between 200 and 300 nanometers have a periodicity on a scale comparable with the wavelength of visible light, they exhibit plentiful beneficial crystal properties for photon propagation that are typical for electrons, such as bands and band gaps [4,9,10]. However, for the described properties of colloidal crystals, two properties are crucial: the uniformity of silica spheres and their diameter. Only homogeneous spheres can create long-range order, which provides these unique optical properties, while the diameter is responsible for the observed colors [9].

Small silica particles are also characterized by large specific surface areas reaching even hundreds of square meters per gram [11,12,13]. In this form, the material can host a significant amount of guests (nanoparticles, functional groups). This allows the fabrication of nanocomposites which can be used as nano- or mesoscale tools. Such use of spherical silica particles leads to their various potential applications in adsorption, ceramics, catalysis, and drug delivery systems [14,15,16,17,18,19,20,21]. Moreover, by utilizing the large surface area of small particles and adsorbing numerous active units, we can reduce the substrate to a functional agent ratio, as opposed to using macroscopic glass substrates. In addition, the diameter of the substrates and, therefore, the curvature of its surface can strongly affect the properties of the obtained material. The availability of homogeneous spherical silica substrates with diameters that can be tuned to suit needs is extremely important in many fundamental and applied research areas.

As shown above, for the practical use of spherical silica mesoparticles, the crucial factors are the control of the diameter and homogeneity of the size. For this reason, an easy, efficient, and reproducible synthesis method of uniform spherical silica particles, which are the building blocks of synthetic opals, becomes significant. Although the interest in silica spheres has been ongoing for several decades, a great deal of work is still being produced on the parameters of syntheses. As a result, there are still conflicting reports on some details of the silica sphere formation process. Therefore, there is a need for experimental confirmation of the procedures reported in the literature and a clear systematization of this topic.

This paper is devoted to reviewing the available synthesis routes for spherical silica particles, their testing and verification (selecting the most efficient ones), and their correction and optimization. Finally, we compiled them in such a way that the reader can plan the synthesis of silica spheres with assumed diameters in the range of 50 nm to 550 nm. Importantly, we ensured that the diameter distribution of the obtained particles would be as narrow as possible.

There are many approaches to the synthesis of silica nanoparticles [22,23]. However, in our opinion, the most promising family of methods is based on the Stöber procedure, which requires only four reagents and results in particles of uniform shape and size [24]. During the optimization of this method (and other derivative procedures as well), we focused on the effect of temperature and concentration of reagents on the morphology of the synthesized silica particles. In particular, our goal was to find parameters that would allow us to reliably obtain particles with diameters of 50, 100, 150, 200, 250, 300, 350, 400, 450, and 550 nm. Intermediate sizes can be interpolated based on the provided procedures.

While developing the procedures for silica spheres with the provided diameters, we encountered a lot of misleading information in the literature. The lack of clear guidelines and simple, reliable solutions prompted us to share our experiences in this area.

## 2. Results and Discussion

The samples were synthesized according to four synthesis procedures, based on the Stöber method [25]. Each series of samples was prepared for different temperatures ranging from 30 to 80 °C, with a step size of 10 °C.

The procedures differ in the molar concentrations of the reagents. For details, see the Section 3.1. Samples were named A-T, B-T, C-T, and D-T, where T refers to the reaction temperature. The molar concentrations of the components and sample designations are listed in Table 1.

Just after the synthesis, each sample series was characterized regarding shape, diameter, and uniformity. This was carried out by means of dynamic light scattering and microscopic observations, using TEM for samples with a diameter below 80 nm and SEM for samples with a diameter above 80 nm.

The microscopic observations allowed us to assess the shape and size of particles directly. We analyzed the shapes of individual particles separately, taking under consideration at least 300 silica spheres, to obtain correct averaging.

DLS provides an effective way to determine the average particle size and size distribution of spherical particles dispersed in suspensions of controlled pH, ionic strength, and temperature. We can treat this technique as a bulk analysis since a large sample volume is measured. Thus, obtained results are averaged from a large population.

Here, we must remark that the DLS tends to show larger particle sizes. Such a technique measures the hydrodynamic diameter of the particle. In practice, large particles suspended in solutions are not spherical—they are dynamic (moving) and solvated. Therefore, the diameter calculated from the diffusion properties of the particle indicate the apparent size of the dynamically solvated particle. For this reason, we chose the results obtained from the micrographs to select the most appropriate method. DLS was used as a complimentary method and a preliminary assessment of the behavior of the particles in suspensions. However, the stabilization of suspensions by any means was not the subject of this work. Table 2 presents the parameters obtained from both approaches. The particle size measured by DLS differs slightly from the particle size estimated from electron microscopy observations, as DLS analysis is based on the behavior of particles in a liquid, which is an environment where particles prone to agglomeration. Nevertheless, the hydrodynamic diameters remain in good agreement with the results obtained from the microscopic observations. Also, the literature analysis confirmed the correctness of the obtained results [24,27].

On the basis of the results shown in Table 2, we chose the most promising sample series—the most promising synthesis routes for the preparation of silica spheres with selected diameters. We aimed for the diameters of 50 nm, 100 nm, 150 nm, 200 nm, 250 nm, 300 nm, 350 nm, 400 nm, 450 nm, and 550 nm as the representative samples required for the most common applications and research. As the most effective procedures to obtain mentioned spherical particles, we selected the following procedures: A-80, A-60, A-50, A-40, B-50, D-40, B-40, C-50, B-30, and C-30, respectively, and those methods are marked with a bold font in Table 2. These samples were selected for further analysis.

**Figure 1:**
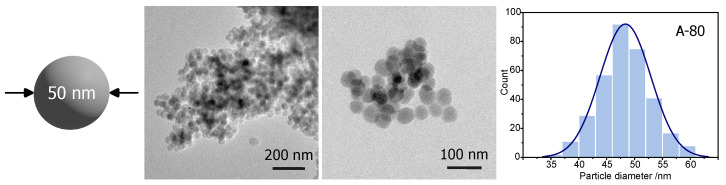
TEM micrographs of the A-80 sample with a diameter of approximately 50 nm, and a histogram presenting the distribution of the particle diameters.

Procedure A-80 was selected as the most efficient for fabricating silica spheres with a diameter of 50 nm. Samples obtained with this synthesis route were observed by TEM. The results can be seen in Figure 1, along with a histogram of the measured diameters.

The synthesis resulted in (middle columns in Figure 1) particles of rather spherical shape and a homogeneous diameter, with an average value of 47 ± 4 nm (histogram—right column in Figure 1). The calculated standard deviation among spheres’ diameter was only 4 nm. This observation agrees with DLS measurements, showing the average diameter of spheres of 55 ± 12 nm. Thus, procedure A-80 seems to be efficient for fabricating spherical silica particles with a diameter of approximately 50 nm.

**Figure 2:**
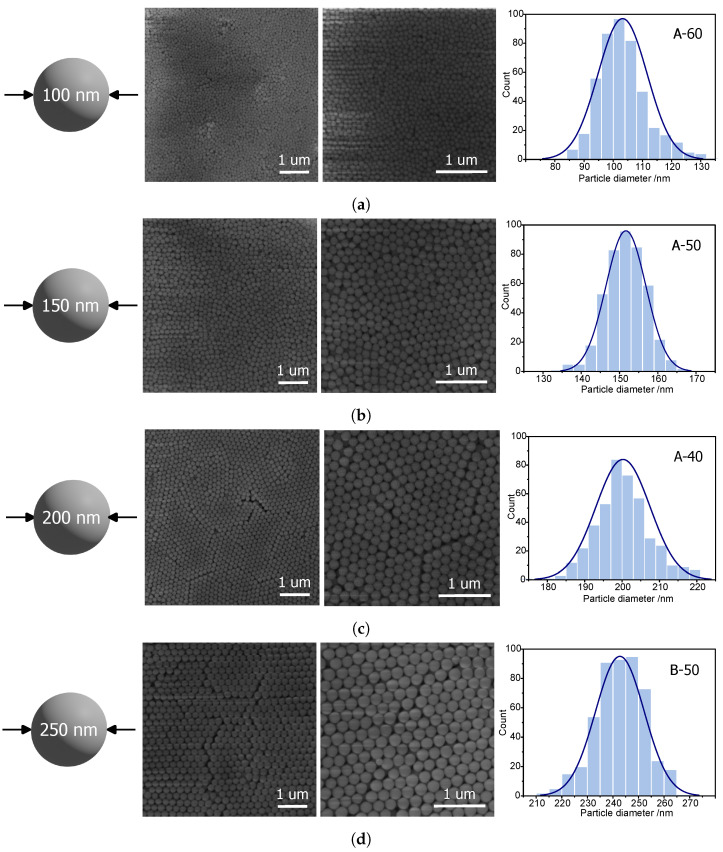

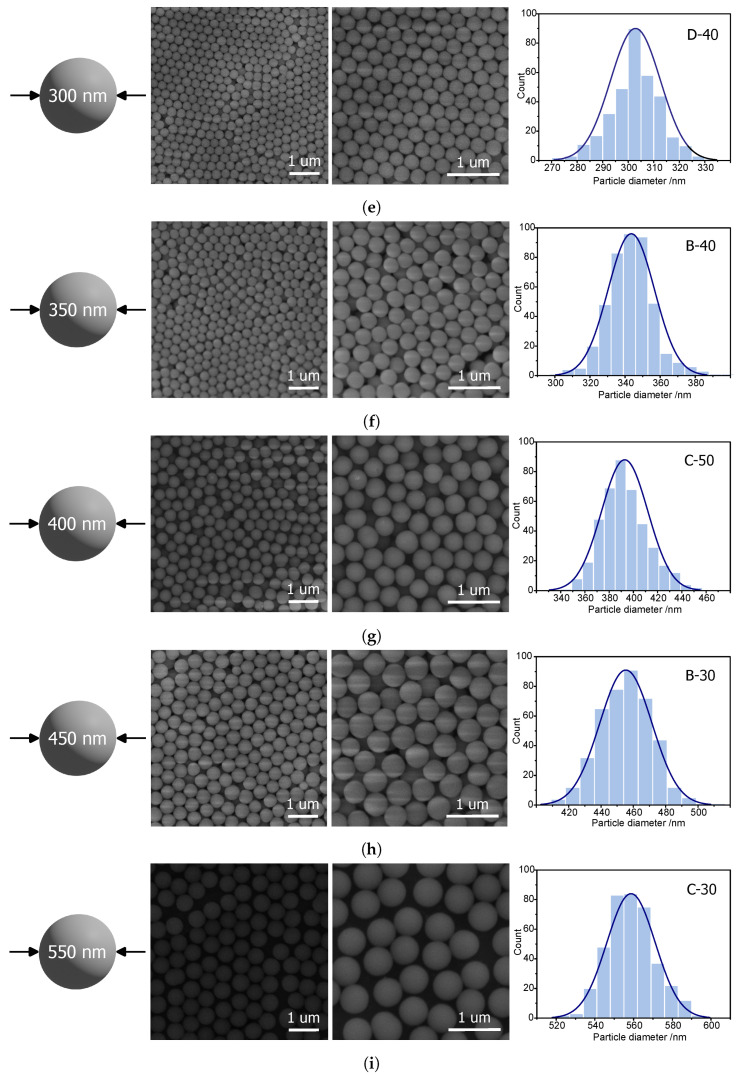
SEM micrographs of samples representing the diameters closest to 100 nm (**a**), 150 nm (**b**), 200 nm (**c**), 250 nm (**d**), 300 nm (**e**), 350 nm (**f**), 400 nm (**g**), 450 nm (**h**), and 550 nm (**i**) (two middle columns), with histograms presenting the distribution of the particle diameters (right column). The target diameters are presented in the left column.

The remaining samples were observed using scanning electron microscopy. In this case, the diameters were sufficient for accurate shape analysis of silica spheres. The microphotographs can be seen in Figure 2.

Spherical silica with a diameter of approximately 100 nm can be most efficiently obtained via the A-60 procedure. SEM pictures (Figure 2a) reveal quite homogeneous spheres. The average diameter of the silica spheres can be estimated as 103 ± 8 nm (Figure 2a—right). A higher value is given by DLS—112 ± 5 nm. Thus, the A-60 procedure can be considered effective for producing spherical silica particles with a diameter of 100 nm.

The 150 nm silica spheres, in turn, can be efficiently synthesized by means of the A-50 procedure. The SEM image (Figure 2b) shows homogeneous, spherical particles with an average diameter of 151 ± 5 nm (see: histogram in Figure 2b). Also, in this case, the DLS measurements give a slightly higher value of 166 ± 6 nm.

Similarly, 200 nm silica spheres can be reliably fabricated, this time via the A-40 procedure. Also, in this case, the homogeneity is quite good (standard deviation of 7 nm), and the average diameter is exactly like the assumed one, 200 nm, as can be seen in the histogram in Figure 2c. Moreover, SEM images show the hexagonal ordering of the spheres which additionally proves the uniformity of the obtained material. The DLS measurement shows a larger value (211 nm).

Similar silica spheres can be synthesized with the C-80 procedure. Here, the average diameter is 205 ± 12 nm, confirmed by DLS, giving 215 ± 3 nm as the average value. However, better results were obtained with the procedure described above.

To obtain particles the closest to 250 nm, the most efficient seems to be the B-50 procedure. Similarly to the previously described material, this sample also presents uniformity, allowing for the hexagonal ordering of silica spheres and the correct spherical shape of particles (Figure 2d). The average diameter of the particles was calculated as 242 ± 9 nm (Figure 2d). Surprisingly, DLS measurements provided a smaller value of the diameter (218 ± 12 nm). Such a result is difficult to explain.

For 300 nm spheres, we chose the D-40 procedure, which resulted in spherical silica particles with an average of 302 ± 10 nm, thus quite close to the assumed value. The result obtained from the DLS calculation was a little larger (324 ± 7 nm), yet was acceptable for the reason described above (Figure 2e).

Procedure B-40 resulted in spheres with average diameters of 344 ± 13 nm for the assumed 350 nm. The SEM pictures (Figure 2f) show the hexagonal ordering of the spheres, proving good homogeneity of the shapes. DLS results indicated an average diameter of 359 ± 4 nm.

The 400 nm silica spheres could be obtained by the C-50 procedure, resulting in 393 ± 19 nm silica spheres (Figure 2g). DLS, in turn, showed a slightly smaller value of the diameters (389 ± 26 nm).

Quite close to the assumed 450 mn were the samples obtained from the B-30 procedure. We obtained homogeneous silica spheres with an average diameter of 455 ± 16 nm, which were capable of hexagonal ordering (Figure 2g). A similar diameter (466 ± 12 nm) was evaluated by DLS measurements.

Obtaining 500 nm silica spheres was impossible for temperatures of 30, 40, 50, 60, 70, and 80 °C. However, we obtained sizes close to 550 nm (558 ± 13 nm) from procedure C-30. The obtained results of the SEM observations were in good agreement with the DLS measurement, this time resulting in sizes of 561 ± 19 nm.

**Figure 3:**
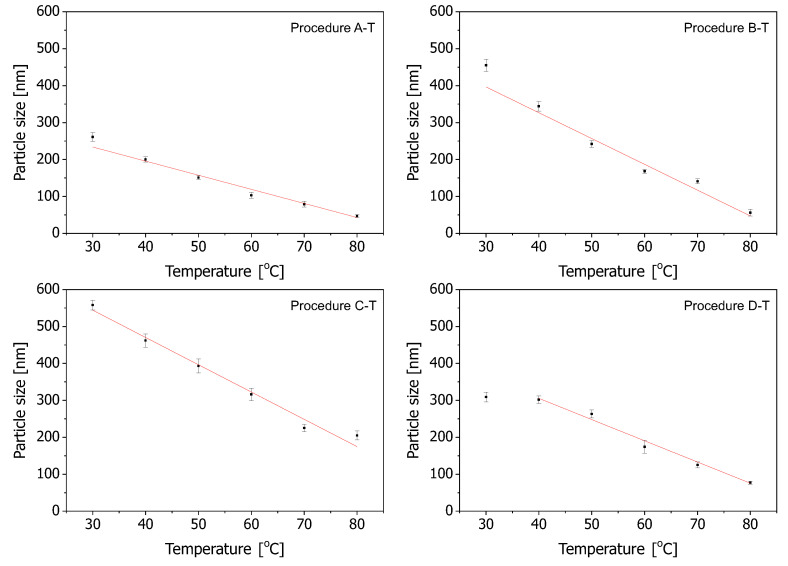
The influence of the reaction temperature on the particle size. The red lines represent the linear fitting of the plotted points. For the case of the D-T procedure, the lowest temperature was excluded from the linear fitting due to apparent inconsistency.

In order to look closer at the dependence of the spherical silica diameter on the reaction temperature, we plotted it in Figure 3 for all the procedures, along with a linear fitting. Here, we must remark that the lowest temperatures (30 ${}^{\circ }$C) in the D-T procedure were not taken into account for linear fitting since, for this temperature, the resulting spherical silica sizes seemed to be questionable—the assigned point seemed to be much too low on the graph.

For the analysis, we took the sizes calculated on the basis of SEM observations.

As can be seen, the smallest slope of the linear regression curve can be attributed to the A-T procedure (the upper-left plot in Figure 3). In this case, changing the reaction temperature affects the diameter of the resulting spheres to a limited extent. Quite a similar situation can be observed for the D-T procedure (the lower-right plot in Figure 3). However, in this case, the slope is a little more narrow. The most narrow slopes of the linear regression curve can be observed for B-T and C-T procedures (the upper-right and lower-left plots in Figure 3, respectively). The C-T procedure covers the widest range of the diameters of silica spheres and can be considered the most efficient with regards to obtaining a wide range of sizes of silica particles.

To summarize this stage of the research, we list the procedures resulting in particle sizes in the range of 50 nm to 550 nm, increasing in 50 nm steps, in Table 3.

### 2.1. Physisorption Analysis

**Figure 4:**
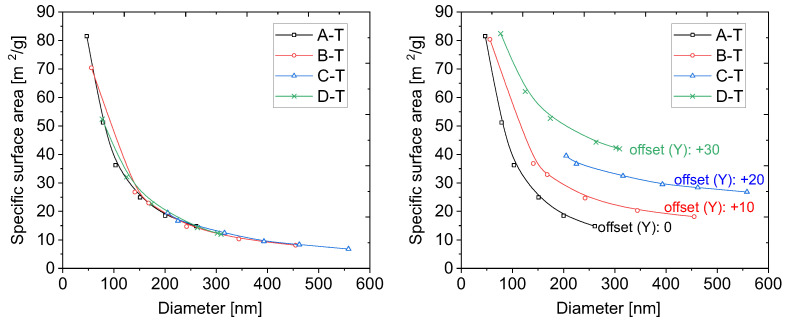
The dependence of the specific surface area on the diameter of the particles. The left plot presents the native results, connected with the B-spline curve, while the right plot shows the same results presented with an offset in the Y-axis for better visibility.

In order to evaluate the specific surface area, the physisorption of nitrogen technique was applied. We plotted the specific surface area of the samples as a function of their diameter in Figure 4. The size of the diameters was taken from microscopic measurements.

The experimental points in the plot (Figure 4) seem to follow the 1/x line. Moreover, the shapes of the plots are almost identical for all the series. This proves that the structure of the particles, obtained with various methods, is the same. The results presented above seem to be obvious and trivial. However, they lead to interesting conclusions, as we present below.

We can draw the theoretical dependence of the specific surface area on the diameter of the silica particles. Specific surface area can be defined as the quotient of the surface area of a single silica sphere by its mass. Taking into account the dependence of the mass of an ideal single sphere on its volume and density, we obtain the following relationship:

(1) $$S_{theor.}=\frac{6}{\rho \cdot \phi }$$

where ρ is the density of silica and ϕ is its diameter. Latest communications provide the density of amorphous silica as between 1.90 and 2.20 g/cm^3^ [28,29]. We took the average of those two values, 2.05 g/cm^3^, and used it in the Equation (1). This way, we could directly calculate the theoretical value of a specific surface area for various diameters of silica particles, as presented for the selected diameters (close to the size of 50 nm–550 nm, with a 50 nm step) in Table 4.

Interestingly, the obtained values are significantly lower than those obtained from our experiments. The theoretical value from our calculations is on average 79% of the experimental value. The only possibility is an incorrect assumption of the density of silica spheres.

**Figure 5:**
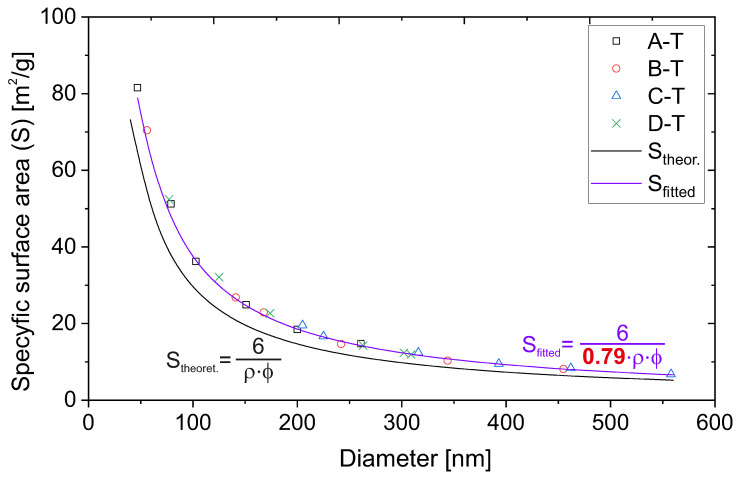
The dependence of the specific surface area on the diameter of the particles obtained, juxtaposed with theoretical dependencies: native and fitted.

The same calculations applied for points taken more densely allow for plotting the dependence of the theoretical specific surface area on the particle diameters. We juxtaposed the obtained curve with experimental points (Figure 5).

We carried out simple fitting with regard to the coefficient *c* at the density value, as in Equation (2):

(2) $$S_{theor.}=\frac{6}{c\cdot \rho \cdot \phi }$$

As can be clearly seen in Figure 5, the perfect fitting was obtained for coefficient *c* equals 0.79, as calculated from Table 4.

**Figure 6:**
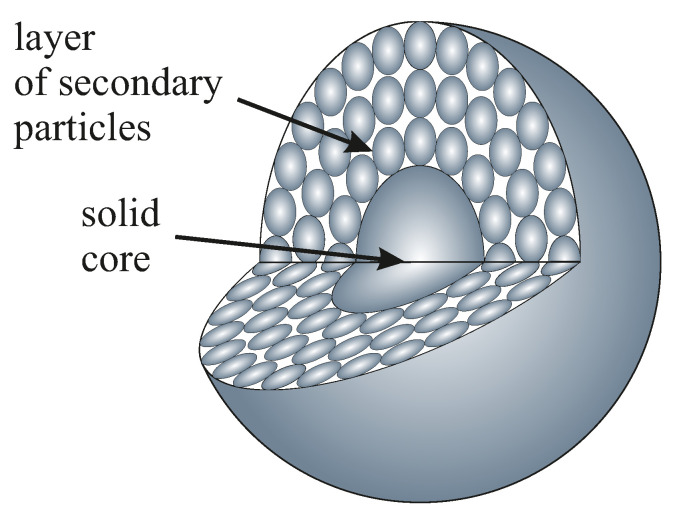
A schematic representation of the two-layer model of the silicon dioxide particles.

Considering the differences between experimental and theoretical values of the specific surface area, we can refer to the assumed intrinsic structure of spherical particles of silica, which still remains unclear. Some authors, however, claim that silica spheres can be composed of a solid central core surrounded by a layer of secondary smaller particles, as can be seen in Figure 6 [15,30]. Such a structure is very difficult to confirm since it is technologically impossible to make an FIB section of a single silica sphere: there is no way to effectively immobilize such a sphere for electron beam cutting.

The internal secondary spheres are closely packed. In the case of both hexagonal and cubic close-packing structures, the volume occupied by spheres is 0.74 of the volume of the elemental cell [31,32]. Assuming the existence of a solid core with an unknown diameter, the volume occupation of 0.79 seems to be probable. Thus, the actual density of silica beads can be 79% of the density value of amorphous silica.

At this point, we should emphasize one important fact. The coefficient *c* at the density value is independent of the silica spheres’ diameter (see the fitting curve in Figure 5). Taking into account the considerations above, we can conclude that the solid core in silica spheres occupies the same percentage of the volume, regardless of their diameter, and it is 5%, as we can easily calculate. Moreover, we can calculate the diameter of the solid core in silica spheres, unifying the total volume of a silica sphere and taking the volume of the solid core as 5% of this volume. After comparing the obtained radii, we have a core radius of 36.88% of the total radius of the silica sphere.

Here, we must again emphasize that the solid core’s radius appears to be the same for different diameters. Given that each synthesis takes the same amount of time, it can be pointed out that the solid core is formed for the same part of the synthesis time during the reaction. After this time, the secondary spheres are created.

**Figure 7:**
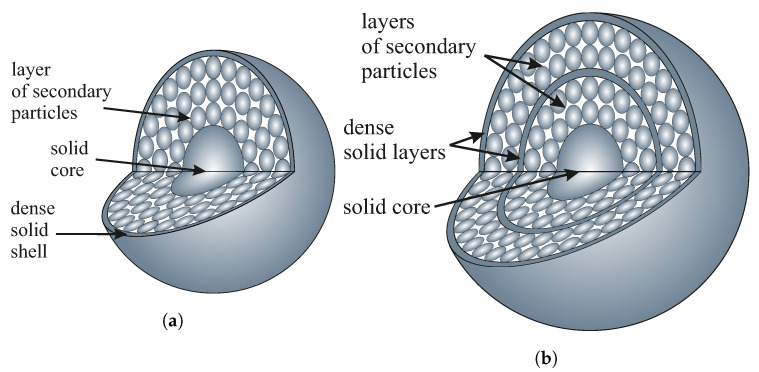
A schematic representation of the shell-like models of the silicon dioxide particles: a single layer of secondary beads (**a**), and multiple layers of secondary beads (**b**).

On the other hand, the considered hierarchical model may imply some additional specific surface area associated with voids between the inner spheres. The specific surfaces obtained for our materials are typical of solid nanomaterials rather than porous spheres [33]. If the inner secondary spheres were accessible to nitrogen, the specific surface area should increase significantly, but it did not. This fact may be due to the existence of an outer solid shell covering the secondary spheres. Such a layer may prevent nitrogen access during sorption analysis. The model can be even more developed and include multiple layers of secondary spheres covered with solid shells like the one presented by Masalov et al. [34]. We present the mentioned models in Figure 7.

To further analyze the proposed models, we calculated the pore volume using the Barrett–Joyner–Halenda (BJH) method [35]. We have assumed that the pore volume is negligible if there is an outer solid shell. It can also be significant if the spheres are accessible to nitrogen, but in this case, we see that it depends monotonically on the diameter of the silica spheres.

The results obtained were completely surprising to us. The pore volume ranged from 0.03 cm^3^/g to 0.4 cm^3^/g, with no apparent dependence on the diameter of the silica spheres. Such a result, however, can be explained by the mechanism of silica particle formation proposed by Masalov et al. [34]. Depending on the diameter of the silica spheres, the last layer of secondary spheres may be completely covered by the outer shell, completely formed but not covered, or partially formed. Of course, in this case, the claim that the solid silica core occupies 5% of the volume in silica spheres should be corrected to occupy 5% of the volume by the core and shells.

Such a hypothesis, however, requires confirmation with additional measurements made on several batches of samples of large numbers, prepared by selected methods over a wide temperature range and a small step of it. The obtained relation should have a sinusoidal shape. However, due to the considerable scope of such studies, such analysis will be the subject of our further article.

### 2.2. Infrared Spectroscopy

**Figure 8:**
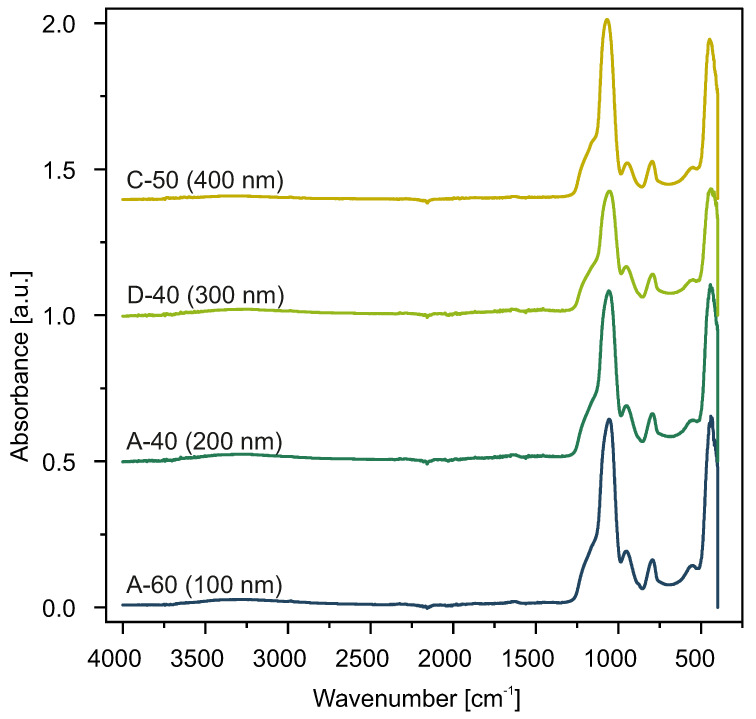
FT-IR spectra of samples A-60 (100 nm), A-40 (200 nm), D-40 (300 nm), and C-50 (400 nm).

FT-IR analysis for all samples revealed very similar features. All spectra exhibit typical silica absorption bands [36]. As an example, we present vibrational spectroscopy results for selected samples obtained via various procedures in Figure 8. The rest of the samples showed almost identical spectra.

**Figure 9:**
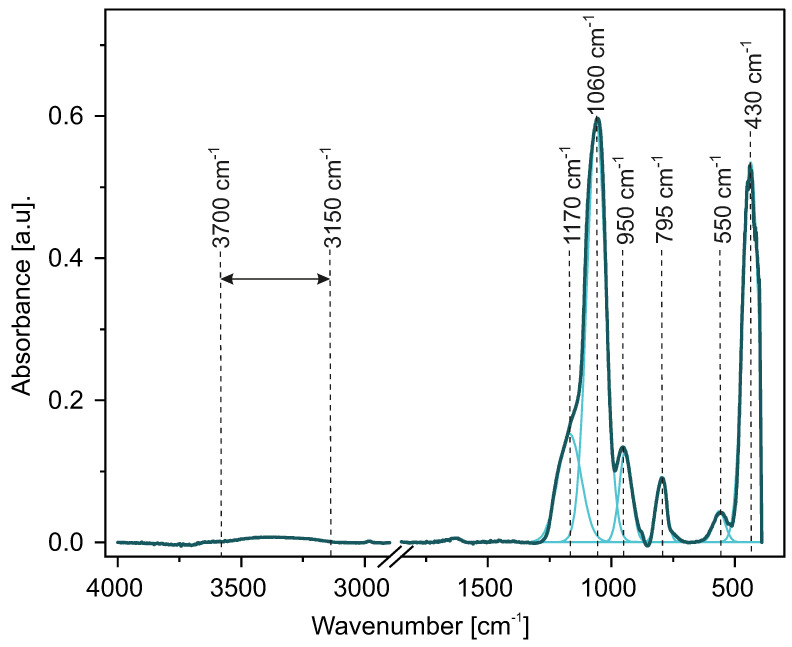
FT-IR spectrum collected for the sample with a diameter close to 100 nm (A-60) after the deconvolution process.

Since all the spectra are similar, we analyzed the A-60 sample as an example. The convoluted characteristic bands of this sample can be seen in Figure 9.

The most dominant band, at 1060 cm${}^{-1}$, is assigned to the asymmetric stretching vibrations of Si–O–Si [37,38]. We also observed bands characteristic to tetrahedron silica structures at 430 cm${}^{-1}$, 550 cm${}^{-1}$, [39], and 795 cm${}^{-1}$ which are assigned to the Si-O bending, Si-O rocking [40,41], and Si-O-Si bending, respectively [42]. The broadband in the region from 3150 to 3700 cm${}^{-1}$ can be attributed to Si-O(H) stretching in silanol groups and adsorbed water [40,42]. Also, the band at 950 cm${}^{-1}$ can be assigned to the Si-O(H) vibrations (bending modes) [40,42].

As can be seen, our samples present all the vibrational modes typical in amorphous silica.

### 2.3. Structural Investigations: XRD and XANES

We examined samples in size range from 50 nm to 550 nm by means of X-ray Diffraction and X-ray Absorption Near-Edge Structure (XANES) to characterize the silica structure.

**Figure 10:**
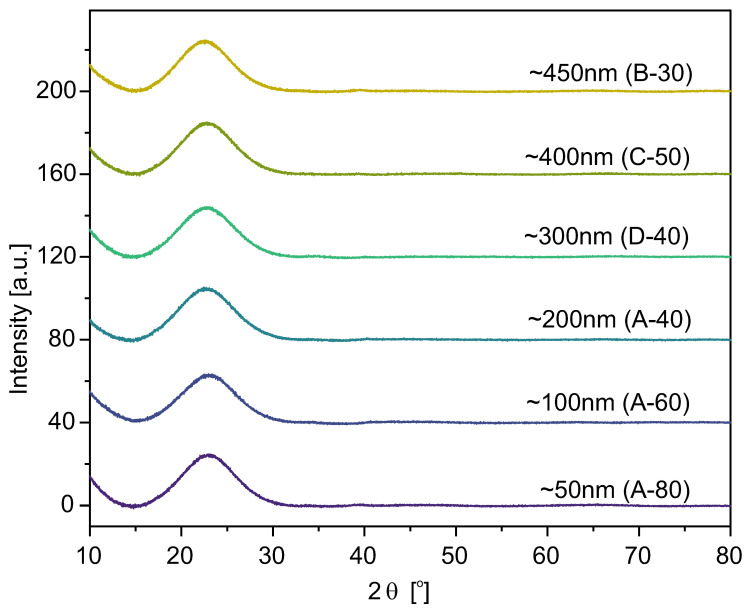
X-ray diffraction spectra of the selected samples.

The X-ray Diffraction spectra for selected samples are presented in Figure 10. For the rest of the samples, the spectra look the same.

The wide bumps on the diffraction spectra for 2θ angles in the range between 15° and 30° were observed for all studied specimens. Furthermore, no sharp diffraction peaks (characteristic of the crystalline phases) were present. Therefore, looking at the plots (in Figure 10), one can conclude that the samples’ structure is typical for amorphous silica, not affected by the synthesis temperature or the particle size obtained [43].

**Figure 11:**
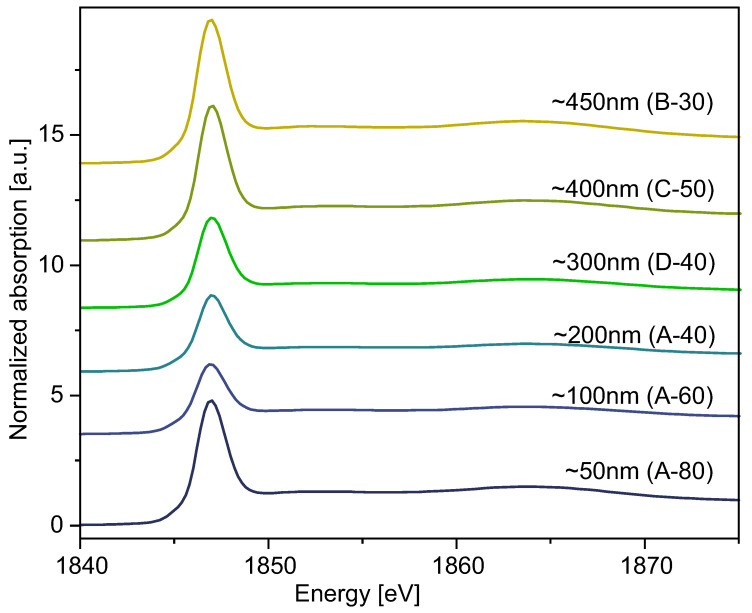
Si K-edge spectra of selected spherical silica samples.

**Figure 12:**
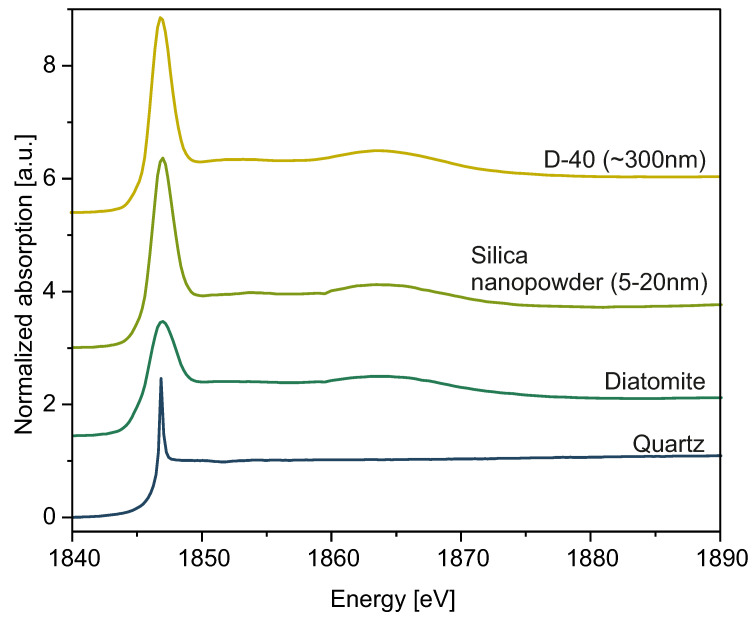
Si K-edge spectra of sample D-40, quartz, diatomite, and commercial silica nanopowder (grain size: 5 nm–20 nm).

XANES spectra of spherical silica particles are presented in Figure 11. For proper interpretation of our results, we used the standard reference samples quartz, diatomite, and commercial silica nanopowder (grain size: 5 nm–20 nm) to compare their spectra measured on the K-edge of Si with spectra obtained for as-synthesized silica particles in sample D-40 (Figure 12). Every data set presented in Figure 11 and Figure 12 was fitted with a Gaussian function and arctan step function, and the results of fitting are collected in Table 5.

Comparing the electrical and local structural characteristics of SiO_2_ in various forms, as represented by spectra collected with XANES spectroscopy, provides essential insights for developing a deeper understanding of the internal atomic arrangement within silica nanospheres. Every spectrum yields an intense white line and absorption peak at E0 = 1846.9 eV, characteristic for amorphous SiO_2_, indicating the 1s—t_2_ (Si 3p–3s) transition [44]. However, the intensities of the peaks varied from one to another. This is why, in order to draw conclusions about the local atomic disorder and electronic properties of the investigated material, we performed data normalization and fitted it with Gaussian and arctan functions to compare the peak widths. Such an approach has been previously employed in the literature [45]. We especially focused on determining the standard deviation of a Gaussian function, σ, which was directly proportional to the full width at half maximum (FWHM) parameter. This value is crucial for determining the widths of peaks, basing on which we inferred the local disorder of the samples. Fitting data, along with the σ values, are presented in Table 5. Differences in the widths of presented peaks between nanosilica powder (NSP) samples and reference samples indicate differences in the local disorder of silica molecules inside investigated materials. Quartz, with the most narrow peak, yields the most crystalline ordering. Diatomite and commercial silica nanopowder show significantly broader peaks, indicating a disordered, amorphous structure. Diatomite, because of its biological nature [46], exhibits the broadest peak, hence indicating the most disordered structure. Commercial silica nanopowder, produced synthetically, has a slightly lower degree of disorder, so its peak is narrower than diatomite’s. Our spherical silica samples exhibit widths comparable to those of commercial SiO_2_ nanopowder. Thus, we conclude the general disordered nature of spheres’ interior structure, albeit to a lesser extent than that presented by diatomite. There are also less intense peaks at approximately 1865.0 eV, which can be seen in the case of every sample other than quartz. The presence of these bumps can be interpreted as multiple scattering imposed on Si electronic structure and possible splitting of 3d orbitals [47,48]. In the case of NSP samples, the spectra do not differ much from one to another, and, other than the 1846.9 eV and 1865.0 eV regions, we did not observe any other peaks, so we can conclude that samples are uniform and amorphous. Moreover, the XANES data obtained may suggest the predominance of the Q^4^ phase of silica [49].

**Figure 13:**
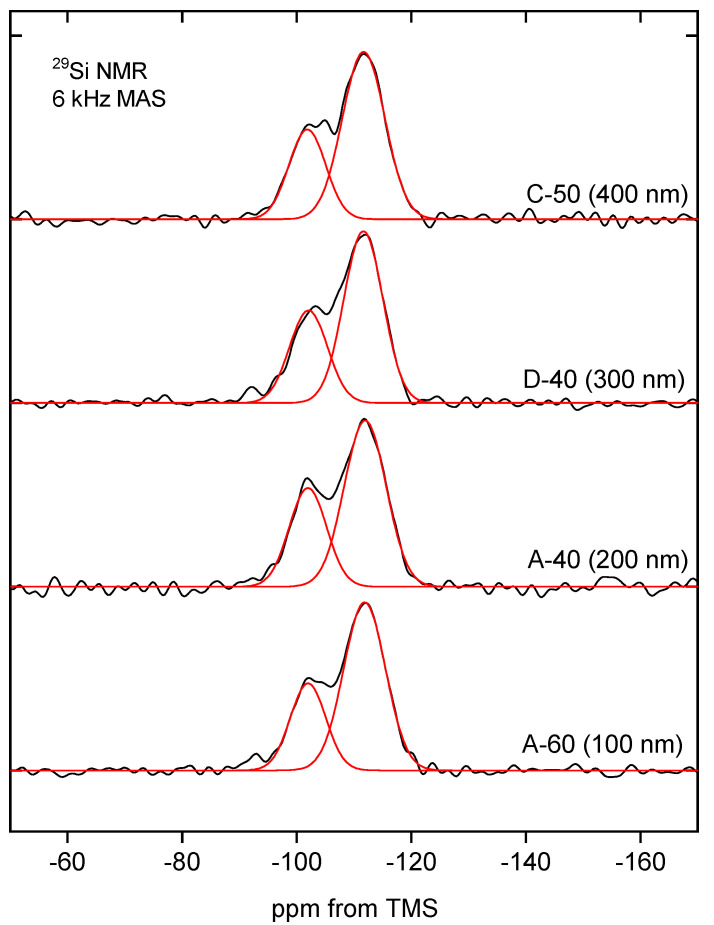
The ^29^Si MAS-NMR spectra of samples A-60 (100 nm), A-40 (200 nm), D-40 (300 nm), and C-50 (400 nm) after the deconvolution process. The black line shows the original spectrum, while the red line is the result of the deconvolution of the spectrum.

Additional information about the molecular structure of the silica building the studied nanospheres was provided by the ^29^Si MAS-NMR. NMR spectra of selected samples can be seen in Figure 13.

The MAS-NMR spectra are quite similar to each other, especially considering the parameters, which are summarized in Table 6. Two intense peaks can be assigned to the silanol groups Q^3^ at −101 ppm and siloxane groups Q^4^ at −111 ppm [50]. The signal from Q^2^ moieties (expected at about −90 ppm) is practically invisible [51].

The relative intensities of the observed peaks indicate that most of the silicon atoms can be assigned to Q^4^ structures and correspond to the silicon atoms incorporated in the silica walls. Q^3^ configurations, also clearly visible on the spectrum but with lower intensity, represent silicon atoms located on the surface and around defects inside the silica structure. Q^2^ substructures can be found mainly on the surface of silica, but their concentration is negligibly low, and the corresponding peak is barely visible in some spectra.

The relatively high proportion of Q^3^ structures in the spectrum may be indicative of a large silica surface area, which, however, was not apparent in nitrogen sorption studies. This, in turn, seems to support the thesis of a hierarchical structure of silica spheres and the isolation of successive layers of secondary spheres by solid shells.

The MAS-NMR spectra indicate that the molecular structure of spherical silica, regardless of the synthesis procedure and particle diameter, is similar.

## 3. Materials and Methods

The scanning electron microscope (SEM) micrographs were recorded at 30 kV using the SE detector of a Tescan Vega 3 scanning electron microscope (Tescan, Brno, Czech Republic). Multiple areas were selected at random for observation. The transmission electron microscopy (TEM) imaging was performed using the FEI Tecnai G2 20 X-TWIN electron microscope (FEI Company, Hillsboro, OR, USA), equipped with emission source LaB${}_{6}$ and CCD camera FEI Eagle 2K (FEI Company, Hillsboro, OR, USA). The diameter size of the spheres were measured from micrographs using ImageJ V1.48 software [52]. No fewer than 300 particles were measured for each sample.

The hydrodynamic diameter (size) of SiO_2_ spheres dispersed in aqueous suspensions was determined using the dynamic light scattering technique (DLS). A Zetasizer Nano ZS (Malvern Instrument, Malvern, Worcestershire, UK) working at a laser wavelength of 532 nm was used for the investigation. The measurement range for particle size was 0.6 nm to 6 μm. For the measurements, the SiO_2_ spheres were suspended in a filtered water solution to obtain suspensions of mass concentration equal to 100 mg/L and pH of approximately 6.5. The measurements were conducted at the temperature of 25 °C. The hydrodynamic diameter of SiO_2_ particles was calculated from the Stokes–Einstein equation [53,54].

Fourier-transform infrared (FTIR) spectra were recorded with the use of a Nicolet İS50 FT-IR spectrometer (Thermo Fischer Scientific, Waltham, MA, USA) in the range 380 cm${}^{-1}$–4000 cm${}^{-1}$ at a resolution of 2 cm${}^{-1}$.

The specific surface area was determined using isothermal sorption of nitrogen. Adsorption–desorption isotherms were recorded at 77.4 K using an Autosorb iQ (Quantachrome Instruments, Boynton Beach, FL, USA) analyzer. The specific surface areas were calculated according to Brunauer–Emmett–Teller (BET) method from the adsorption data in the relative pressure range of 0.14–0.26.

To investigate the degree of disorder of the silica, we used Si K-edge XANES (X-ray Absorption Near Edge Structure). Measurements were carried out in transmission mode at the ASTRA (Absorption Spectroscopy beamline for Tender energy Range and Above) beamline at the NCSR SOLARIS synchrotron in Kraków, Poland. Along with prepared spherical silica samples of different diameters, commercial samples of quartz, diatomite, and SiO${}_{2}$ nanopowder (grain size: 5 nm–20 nm) were used as a reference. Every sample was ground in a mortar (with starch as a filler when needed) until uniform; fine powder was obtained, which was placed on a sample holder between two layers of thin Kapton tape. To cover the energy range around and above the value of Si K-edge, InSb(111) monochromating crystals were chosen for all measurements. All samples were examined at room temperature and in a N${}_{2}$ atmosphere under low pressure of 11 Torr. Data points were collected using dedicated AstraLibra software and later analyzed using the ATHENA package included in the Demeter 0.9.26 package. We also used the Athena software to perform background removal and normalization, and to fit the data for obtaining parameters necessary for drawing conclusions about our samples.

We used high-resolution magic-angle spinning nuclear magnetic resonance spectroscopy (MAS-NMR) as an additional technique to study the internal structure of silica. The MAS-NMR spectra were measured using an APOLLO console (Tecmag, Houston, TX, USA) and a 7 T/89 mm superconducting magnet (Magnex, Oxford, UK). A high-speed MAS Bruker HP-WB probe (Bruker, Karlsruhe, Germany) equipped with a 4 mm zirconia rotor and a KEL-F cap was used to spin the sample.

The ^29^Si MAS-NMR spectra were measured at a resonance frequency of 59.515 MHz, rotating the sample at 6 kHz. A single 3 μs rf pulse was used, corresponding to a flip angle of π/2. The acquisition delay in accumulation was 20 s, and 512 scans were performed. The ppm frequency scale was referenced to the ^29^Si TMS (tetramethylsilane) resonance. Peak matching of the ^29^Si MAS-NMR spectra was carried out using an automated procedure, without making any a priori assumptions about the positions of the lines or their widths. The minimum number of components that yield satisfactory residuals was selected.

Complementary to the aforementioned techniques, X-Ray Diffractometry (XRD) was conducted using a Bruker D8 Advance diffractometer with CuKα radiation (λ = 1.5418 Å) and LynxEye detector, operating at 40 kV and 40 mA. The XRD studies of silica were performed in conventional Bragg–Brentano configuration for the range of 2θ angles from 10° to 85° with a size step of 0.01° and a time step of 5 s. The studied powder samples were compacted within the holders to form disks. Each specimen was subjected to rotation during the measurements to collect data from the entire sample.

### 3.1. Samples Synthesis

For the synthesis of the silica particles, we used only four reagents: tetraethylorthosilicate (Si[OC${}_{2}$H${}_{5}$]${}_{4}$, TEOS, 98%, Sigma-Aldrich, Taufkirchen, Germany), ethanol (C${}_{2}$H${}_{5}$OH, 99.9%, Poch Inc., Gliwice, Poland), ammonium hydroxide solution (NH${}_{4}$OH, concentration of 25%), and deionized water, without further purification.

Each synthesis was carried out under exactly the same laboratory conditions (humidity and temperature) and using the same laboratory accessories. As we checked, each parameter is important to obtain reproducible results. Reagents were heated or cooled to a temperature near the assumed one in the procedure before the synthesis.

The polycondensation was carried out in a water bath under stirring using a magnetic stirrer. A round-bottom flask and a Teflon-coated oval stirring bar were used each time. The stirring speed was 900 rpm to mix the solution well while keeping the top layer of the solution in the water bath.

In order to fabricate uniform spherical particles with diameters from 50 to over 550 nm, we applied syntheses based on Gao’s modification of Stöber method [25]—the solvent-varying method. In this approach, the volume amount of TEOS/NH${}_{3}$/H${}_{2}$O was fixed while the volume of ethanol was variable. This procedure involves hydrolysis and condensation reactions at 60 °C in its original form. We decided, however, to combine the method proposed by Gao et al. with the observations confirmed by Liang et al. [27], who stated that an increase in temperature results in a decrease in the diameter of the obtained particles.

The first syntheses batch assumed the molar concentration of reagents in the solution as TEOS/NH${}_{3}$/H${}_{2}$O 0.19/0.36/3.30 in moles. The ratio of TEOS to ammonia was 1.00:1.91. The modification of the sizes of resulting samples was achieved only by changing the synthesis temperature in the range of 30 °C to 80 °C. The resulting samples were named **A-T**, where T is the reaction temperature. The maximum diameter of silica spheres possible with this procedure was 261 nm.

To obtain larger particles, we decreased the amount of ethanol in the solution, changing the final molar concentrations of reagents to TEOS 0.27 M, NH${}_{3}$ 0.52 M, and H${}_{2}$O 4.68 M. There was, however, no change in the ratio of TEOS to ammonia, which was maintained at 1.00:1.91. Similar to the previous case, we modified the silica size by changing the temperature. The resulting samples were named **B-T**, where T stands for the reaction temperature.

The third series of samples (**C-T**) with molar concentrations TEOS/NH${}_{3}$/H${}_{2}$O equal to 0.47 M/0.89 M/8.07 M yielded even larger particles, depending on the used temperature.

Next, we tested the procedure with a higher ratio of ammonia: TEOS equal to 4.38:1.00, proposed by José-Miguel Zérate-Reyes et al. [26] (samples D-T), where T is the reaction temperature. In this approach, the particles were prepared with 0.23 M TEOS, 1.01 M ammonia, and 18.43 M deionized water. Also, this time, we applied different synthesis temperatures to obtain particles with various diameters.

In each case, the reaction time was two hours. A detailed description of each synthesis route can be found in Supplementary Materials (including Figure S1).

## 4. Conclusions

In this paper, we have tested and optimized the most efficient synthesis routes available for producing spherical silica particles with diameters ranging from 50 to 550 nm. We have provided the synthesis outputs in such a way as to allow the selection of the ideal conditions for the fabrication of spherical silica with the assumed diameter.

The investigated synthesis routes were based on the Stöber protocol as the most efficient and easiest way for obtaining homogenous spherical silica particles.

Microscopic observations have shown a high degree of homogeneity of the particles and their spherical shape. Structural investigations have revealed the identical structure of obtained materials regardless of the applied procedure and diameter of particles.

One of the most important outputs was the confirmation and development of the proposed model of the intrinsic structure of artificial opals. Based on the specific surface area measurements and nuclear magnetic resonance investigation, we make a more probable thesis that the silica spheres comprise a solid silica core and several layers of densely packed secondary silica spheres covered with solid silica shells.

Moreover, we calculated that the solid silica core and shells occupy 5% of the volume of the silica particle, regardless of the synthesis procedure and the diameter of the spheres. This result may be of great importance when analyzing the structure of synthetic opals.

## Figures and Tables

**Table 1:** Molar concentrations applied in the four series of samples.

Series Name	TEOS [M]	NH_3_ [M]	H${}_{2}$O [M]	$\frac{c_{{\mathrm{NH}}_{3}}}{c_{\mathrm{TEOS}}}$	Reference
A-T	0.19	0.36	3.30	1.91	[25]
B-T	0.27	0.52	4.68	1.91	[25]
C-T	0.47	0.89	8.07	1.91	[25]
D-T	0.23	1.01	18.43	4.38	[26]

**Table 2:** Temperature-size dependence. Samples selected for further analyses are marked with bold text. Abbreviation designations: D_MIC_—the average diameter of samples obtained from microscopic observations, SD_MIC_—standard deviation assigned to the microscopic assessment of silica spheres’ diameter, D_DLS_—the average diameter of samples obtained from DLS measurements, SD_DLS_—standard deviation assigned to the DLS assessment of silica spheres’ diameter.

Sample Name	T [°C]	D_MIC_ [nm]	SD_MIC_ [nm]	D_DLS_ [nm]	SD_DLS_ [nm]
A-30	30	261	12	273	6
**A-40**	**40**	**200**	**7**	**211**	**14**
**A-50**	**50**	**151**	**5**	**166**	**6**
**A-60**	**60**	**103**	**8**	**112**	**5**
A-70	70	79	8	91	3
**A-80**	**80**	**47**	**4**	**55**	**12**
**B-30**	**30**	**455**	**16**	**466**	**12**
**B-40**	**40**	**344**	**13**	**359**	**4**
**B-50**	**50**	**242**	**9**	**218**	**12**
B-60	60	168	5	181	5
B-70	70	141	7	187	6
B-80	80	56	9	73	15
**C-30**	**30**	**558**	**13**	**561**	**19**
C-40	40	462	18	510	32
**C-50**	**50**	**393**	**19**	**389**	**26**
C-60	60	316	17	338	7
C-70	70	225	10	241	7
C-80	80	205	12	215	3
D-30	30	309	13	295	23
**D-40**	**40**	**302**	**10**	**324**	**7**
D-50	50	263	11	252	5
D-60	60	174	17	185	2
D-70	70	125	8	133	5
D-80	80	77	4	88	7

**Table 3:** The most efficient synthesis routes with assumed diameters of silica spheres (D). For subscripts, (assumed) means assumed value, (MIC) means the results obtained from microscopic observations, and (DLS) means the result from DLS measurements.

D_(assumed)_ [nm]	Synthesis Procedure	D_(MIC)_ [nm]	D_(DLS)_ [nm]
50	A-80	47	55
100	A-60	103	112
150	A-50	151	166
200	A-40	200	211
250	B-50	242	218
300	D-40	302	324
350	B-40	344	359
400	C-50	393	389
450	B-30	455	466
550	C-30	558	561

**Table 4:** Specific surface area of samples with diameters of approximately ∼50 nm, 100 nm, 150 nm, 200 nm, 250 nm, 300 nm, 350 nm, 400 nm, 450 nm, and 550 nm. The S_theor._ means theoretically calculated value, while S_BET_ is an experimental value.

Sample Name	Assumed Diameter [nm]	Average Diameter [nm]	S_theor._ [m^2^/g]	S_BET_ [m^2^/g]	S_theor._/S_BET_
A-80	50	47	62.27	81.53	0.76
A-60	100	103	28.42	36.21	0.78
A-50	150	151	19.38	24.91	0.78
A-40	200	200	14.63	18.45	0.79
B-50	250	242	12.09	14.65	0.83
D-40	300	302	9.69	12.38	0.78
B-40	350	344	8.51	10.29	0.83
C-50	400	393	7.45	9.47	0.79
B-30	450	455	6.43	8.12	0.79
C-30	550	558	5.25	6.79	0.77

**Table 5:** Peak fitting to XANES experimental data summary. The Gaussian function was used as a fitting function and arctan as a step function.

	A-80	A-60	A-40	D-40	C-50	B-30	Quartz	Nanopowder (Grains 5–20 nm)	Diatomite
**Gauss E0**	8.639	4.769	4.71	5.862	9.178	9.765	0.799	6.384	5.276
**Gauss height**	1847	1846.87	1846.88	1846.88	1847	1847	1846.82	1846.93	1846.93
** σ **	0.829	0.884	0.911	0.801	0.82	0.81	0.181	0.885	1.279
**Arctan E0**	1.084	0.87	0.885	0.944	1.099	1.146	1.094	0.804	0.606
**Arctan height**	1847	1846.87	1846.88	1846.88	1847	1847	1846.82	1846.93	1846.93
**Width**	0.331	0.331	0.331	0.331	0.331	0.331	0.331	0.331	0.331
**R-factor**	0.00948	0.00565	0.00481	0.00262	0.00959	0.00882	0.0246	0.00799	0.0119
**Chi-square**	1.817	0.385	0.324	0.268	2.094	2.127	0.717	0.795	0.611
**Reduced chi-square**	0.0423	0.00895	0.00753	0.00623	0.0487	0.0495	0.0167	0.0185	0.0142

**Table 6:** Parameters of ^29^Si MAS-NMR spectra of samples A-60 (100 nm), A-40 (200 nm), D-40 (300 nm), and C-50 (400 nm). FWHM is full width of the peak at half maximum.

Sample	Peak Position [ppm]	FWHM [ppm]	Relative Intensity [%]
A-60	−111.5	8.7	69
(100 nm)	−101.6	7.3	31
A-40	−111.6	8.6	65
(200 nm)	−101.6	7.7	35
D-40	−111.3	8.0	66
(300 nm)	−101.7	7.8	34
C-50	−111.3	8.7	68
(400 nm)	−101.5	7.6	32

## Data Availability

Data available on request.

## Supplementary Materials

The following supporting information can be downloaded at: https://www.mdpi.com/article/10.3390/ijms241813693/s1. References [24,25,26] are also cited in the supplementary materials.

## Abbreviations

The following abbreviations are used in this manuscript:

DLS	Dynamic Light Scattering
SEM	Scanning electron microscopy
TEM	Transmission electron microscopy
MAS-NMR	Magic-angle spinning nuclear magnetic resonance
TEOS	Tetraethylorthosilicate
TMS	Tetramethylsilane
