# Supplementary figures and images for: Synthetic Opals or Versatile Nanotools—A One-Step Synthesis of Uniform Spherical Silica Particles
Source: Int J Mol Sci. 2023 Sep 5;24(18):13693. doi: 10.3390/ijms241813693 (PMC10530679; doi:10.3390/ijms241813693)

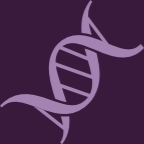

# International Journal of *Molecular Sciences*

Supplement: Supplementary file 1 [file ijms-24-13693-s001.zip › Definitions/ijms-logo-eps-converted-to.pdf]

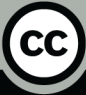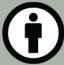

BY

Supplement: Supplementary file 1 [file ijms-24-13693-s001.zip › Definitions/logo-ccby-eps-converted-to.pdf]

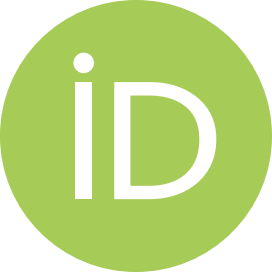

Supplement: Supplementary file 1 [file ijms-24-13693-s001.zip › Definitions/logo-orcid.pdf]

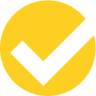

check for  
updates

Supplement: Supplementary file 1 [file ijms-24-13693-s001.zip › Definitions/logo-updates-eps-converted-to.pdf]

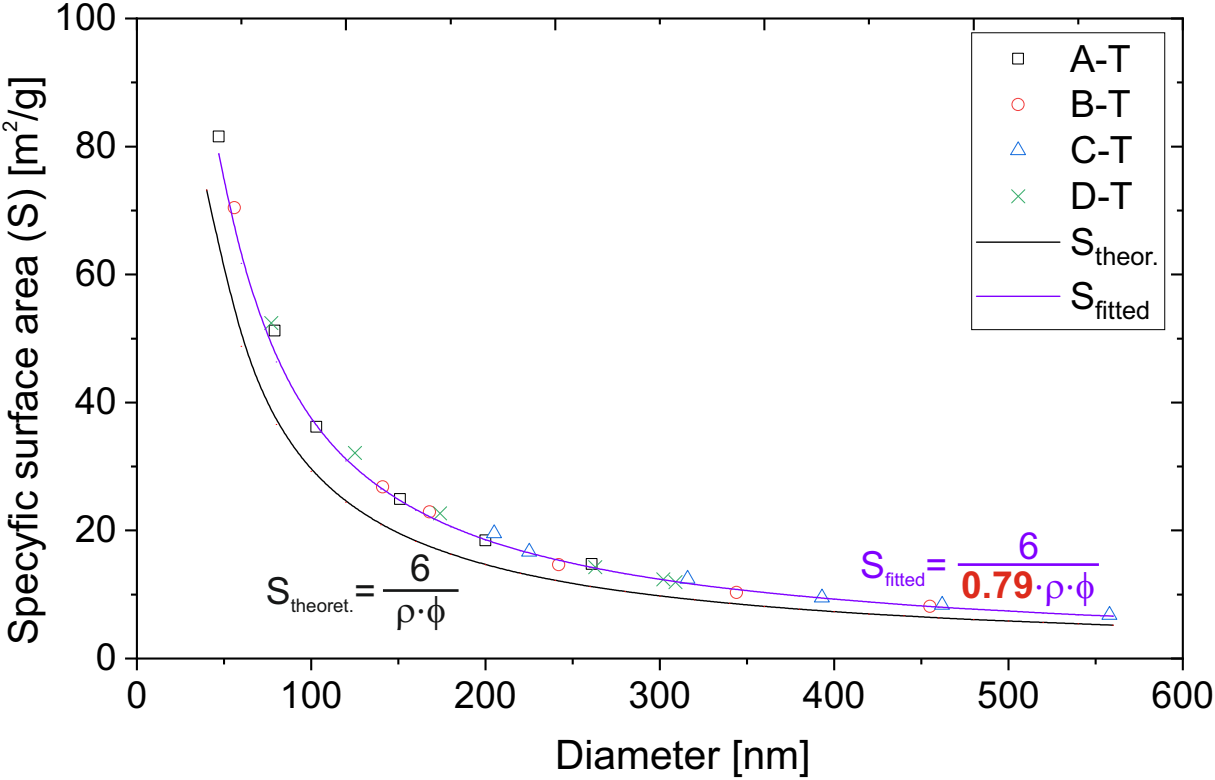

Supplement: Supplementary file 1 [file ijms-24-13693-s001.zip › Figures/BET-fit.pdf]

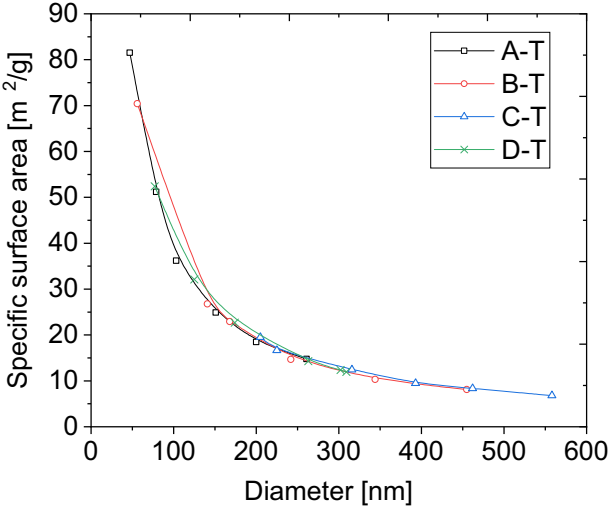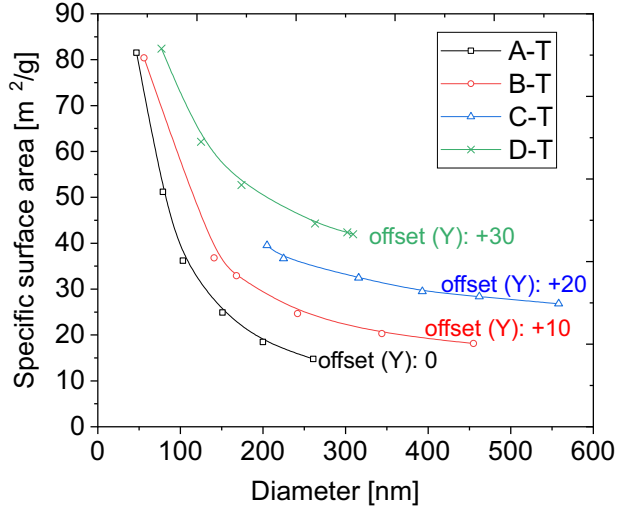

Supplement: Supplementary file 1 [file ijms-24-13693-s001.zip › Figures/BETPlots.pdf]

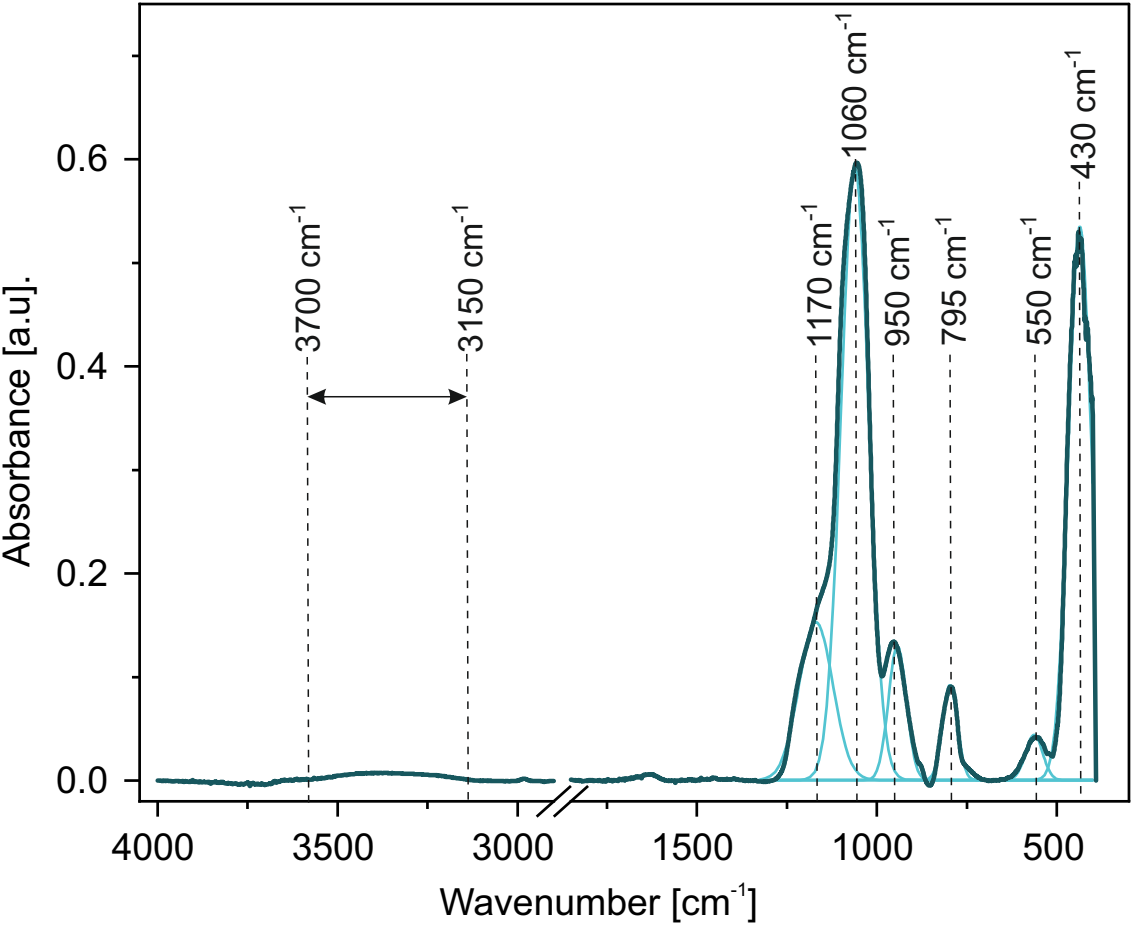

Supplement: Supplementary file 1 [file ijms-24-13693-s001.zip › Figures/FT-IR-Dec.pdf]

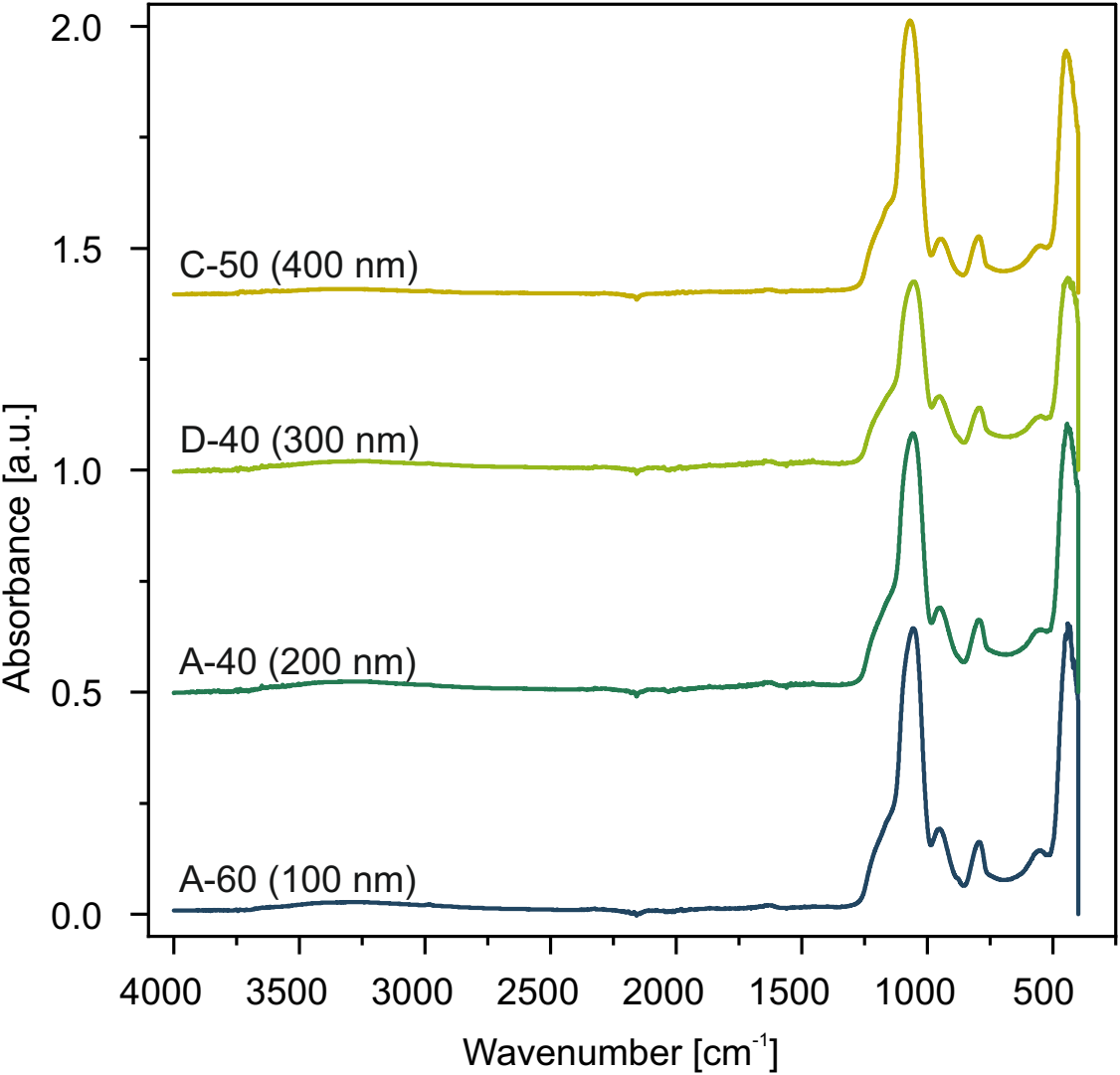

Supplement: Supplementary file 1 [file ijms-24-13693-s001.zip › Figures/FT-IR.pdf]

layer  
of secondary  
particles

solid  
core

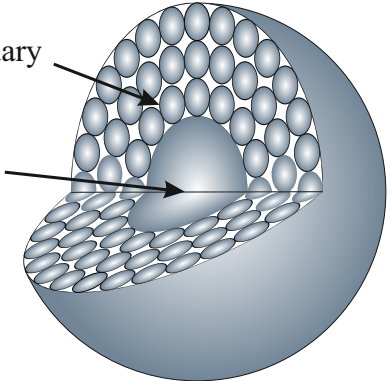

Supplement: Supplementary file 1 [file ijms-24-13693-s001.zip › Figures/IntrStruct.pdf]

layer  
of secondary  
particles

solid  
core

dense  
solid  
shell

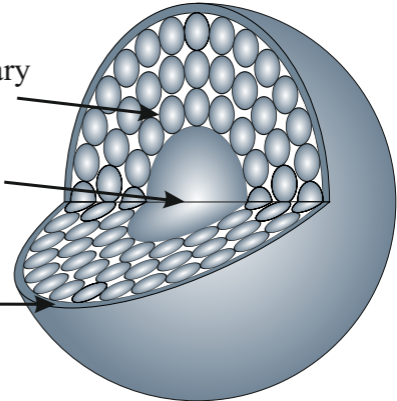

Supplement: Supplementary file 1 [file ijms-24-13693-s001.zip › Figures/IntrStruct2.pdf]

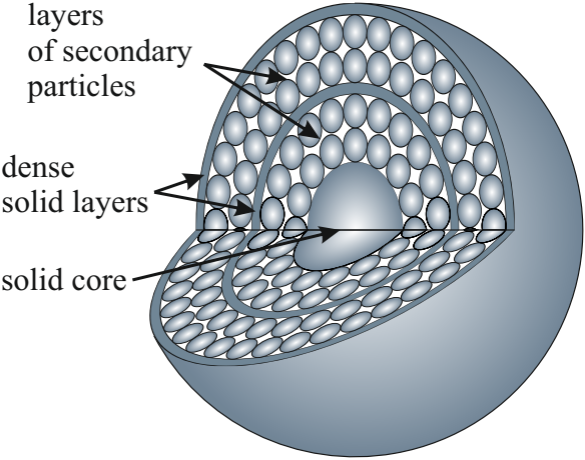

Supplement: Supplementary file 1 [file ijms-24-13693-s001.zip › Figures/IntrStruct3.pdf]

$^{29}\text{Si}$  NMR  
6 kHz MAS

C-50 (400 nm)

D-40 (300 nm)

A-40 (200 nm)

A-60 (100 nm)

-60

-80

-100

-120

-140

-160

ppm from TMS

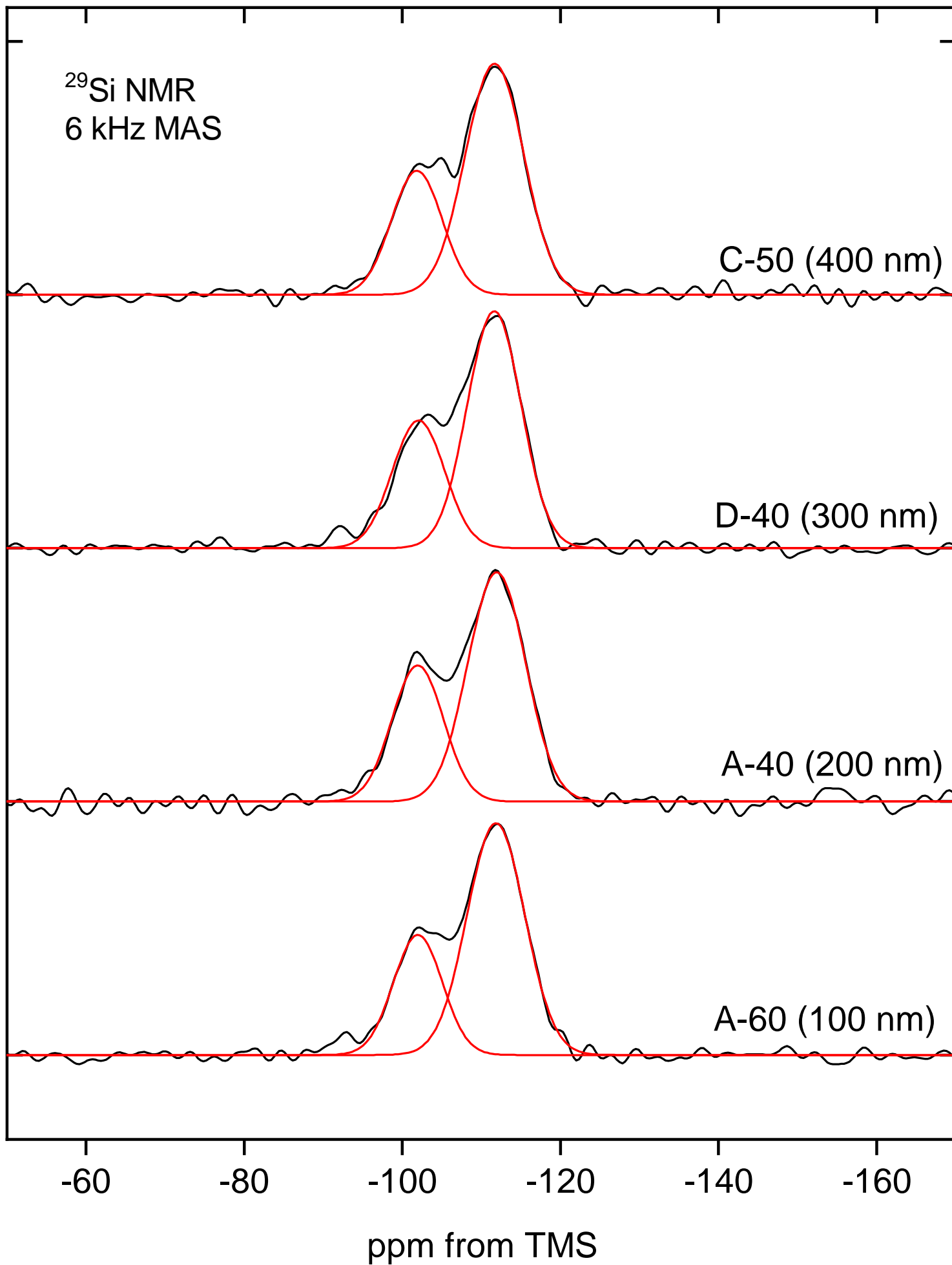

Supplement: Supplementary file 1 [file ijms-24-13693-s001.zip › Figures/NMR.pdf]

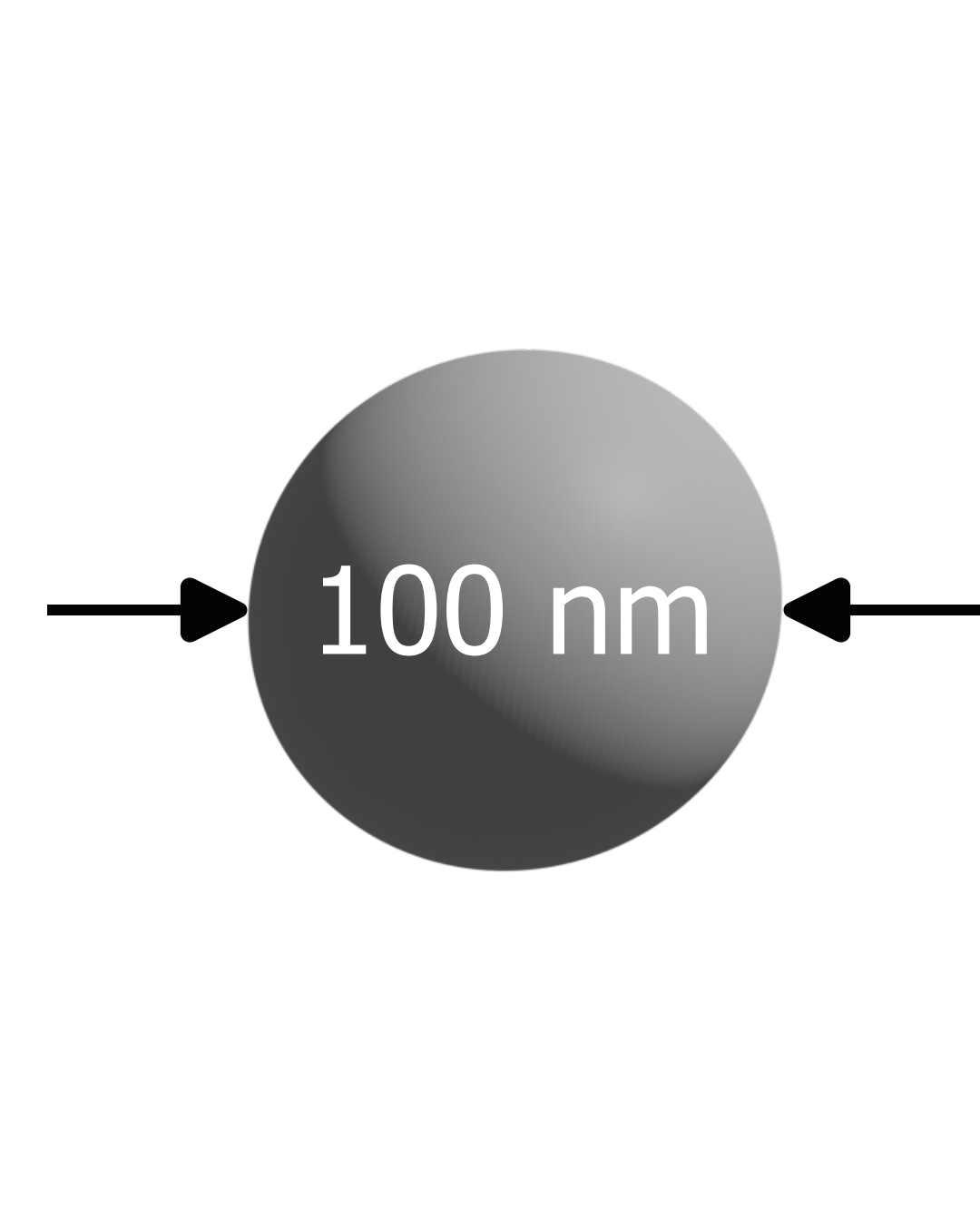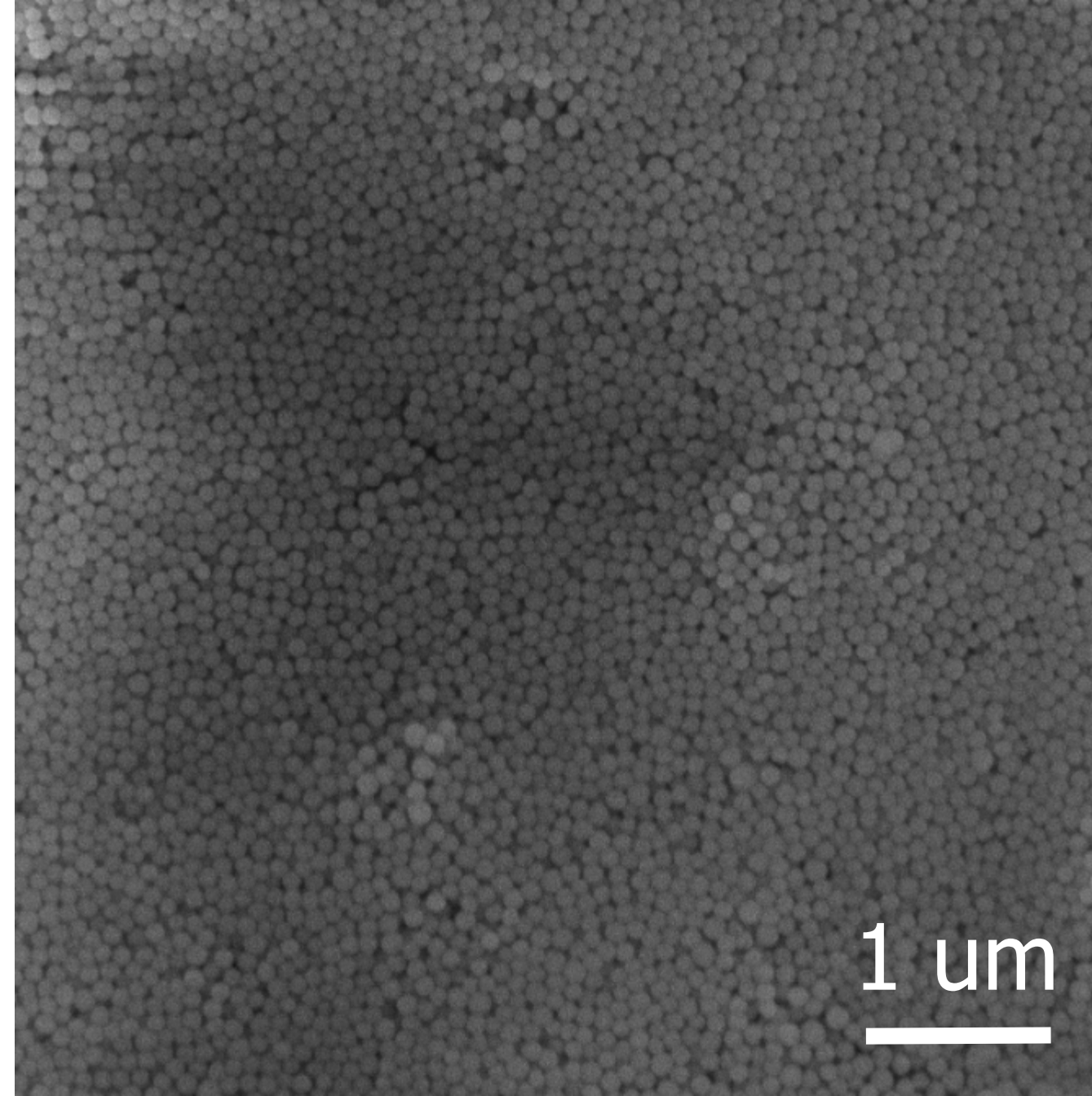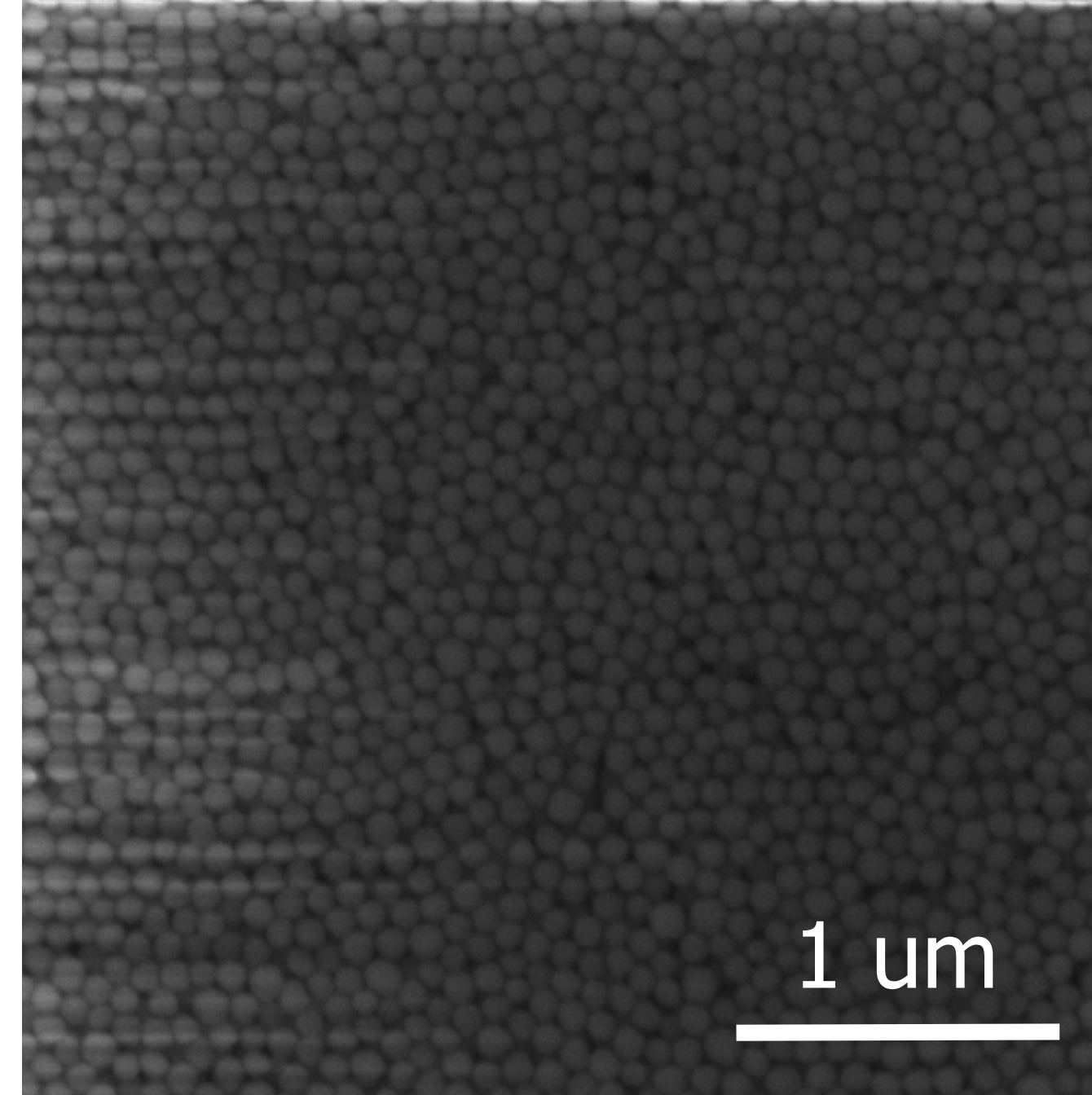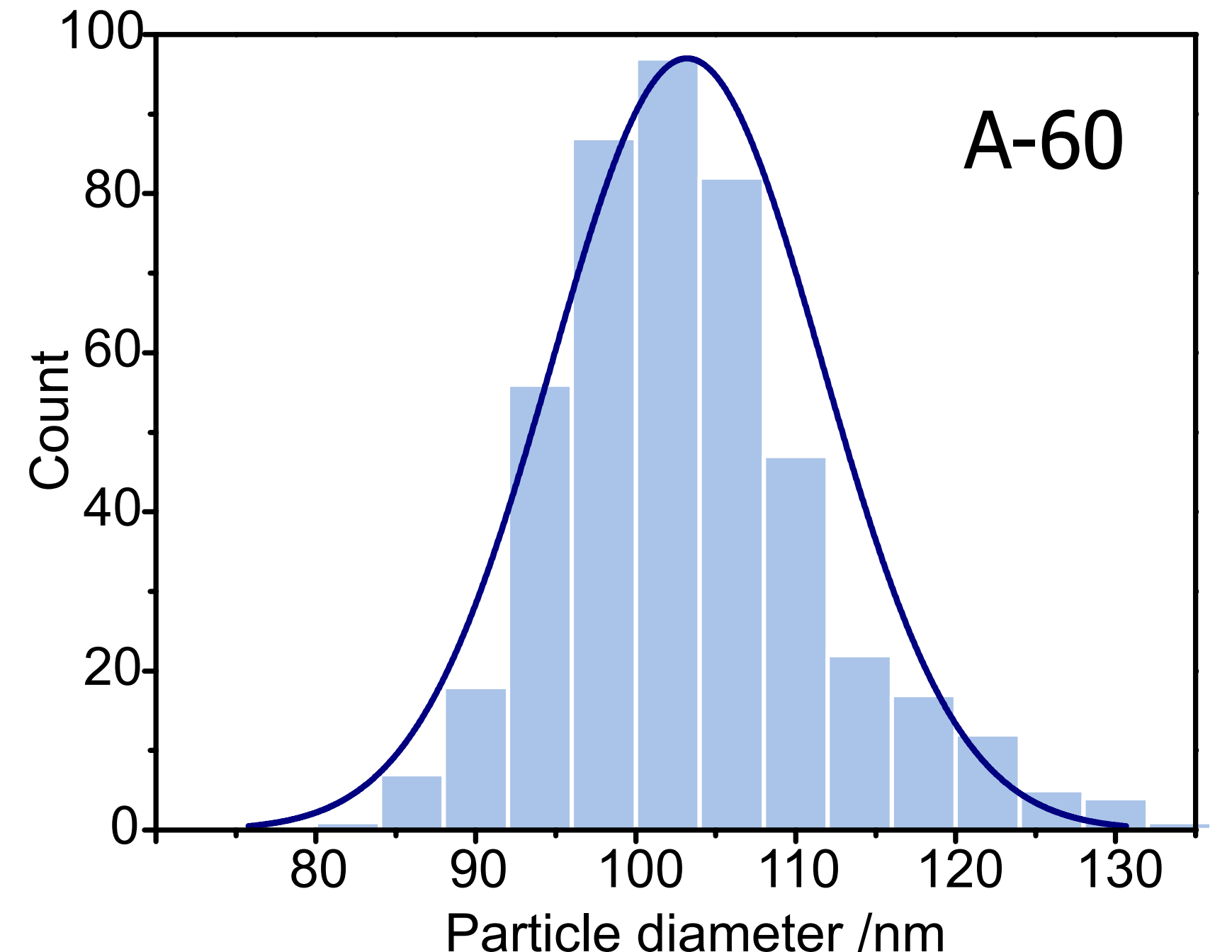

Supplement: Supplementary file 1 [file ijms-24-13693-s001.zip › Figures/SEM/100 nm.pdf]

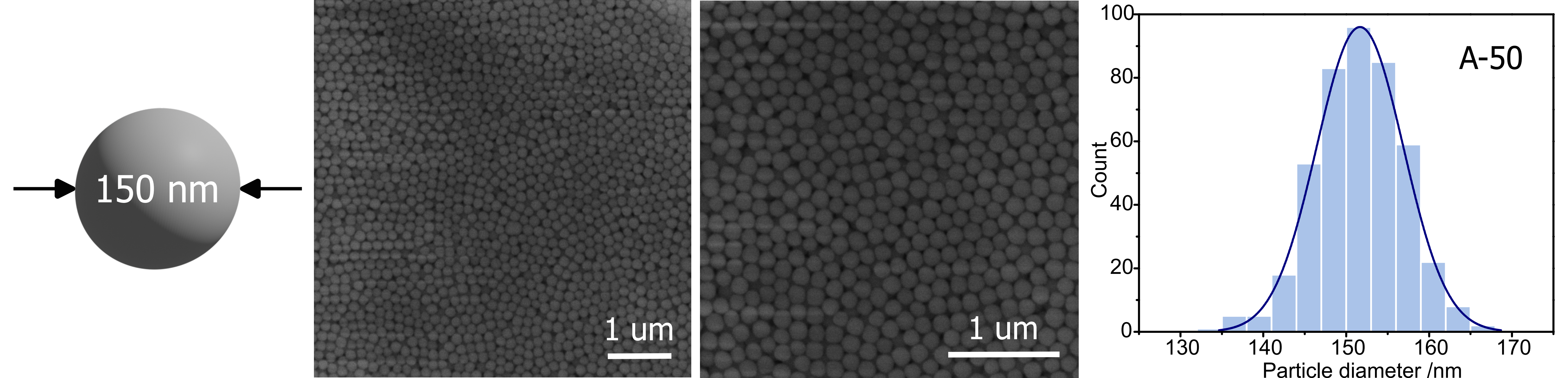

Supplement: Supplementary file 1 [file ijms-24-13693-s001.zip › Figures/SEM/150 nm.pdf]

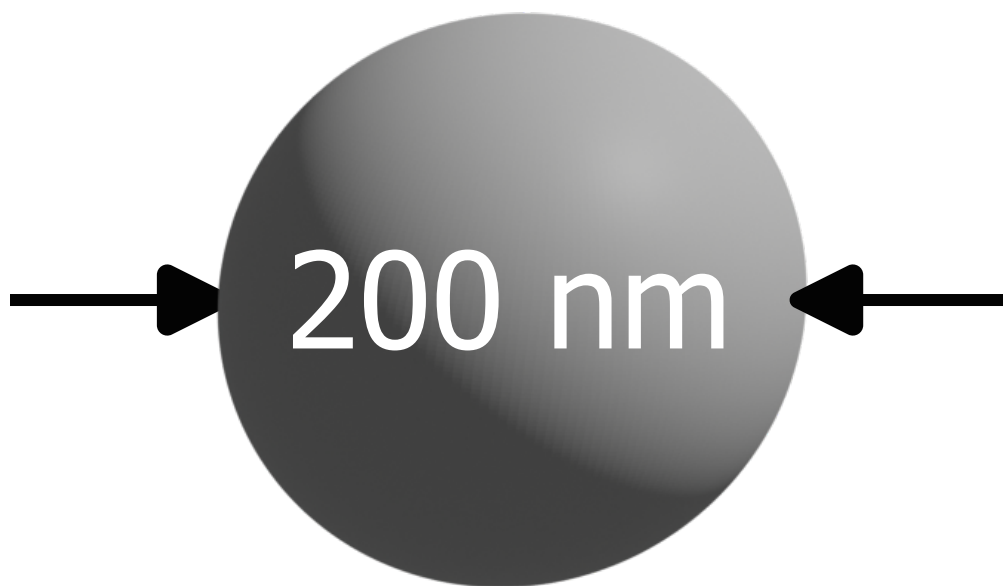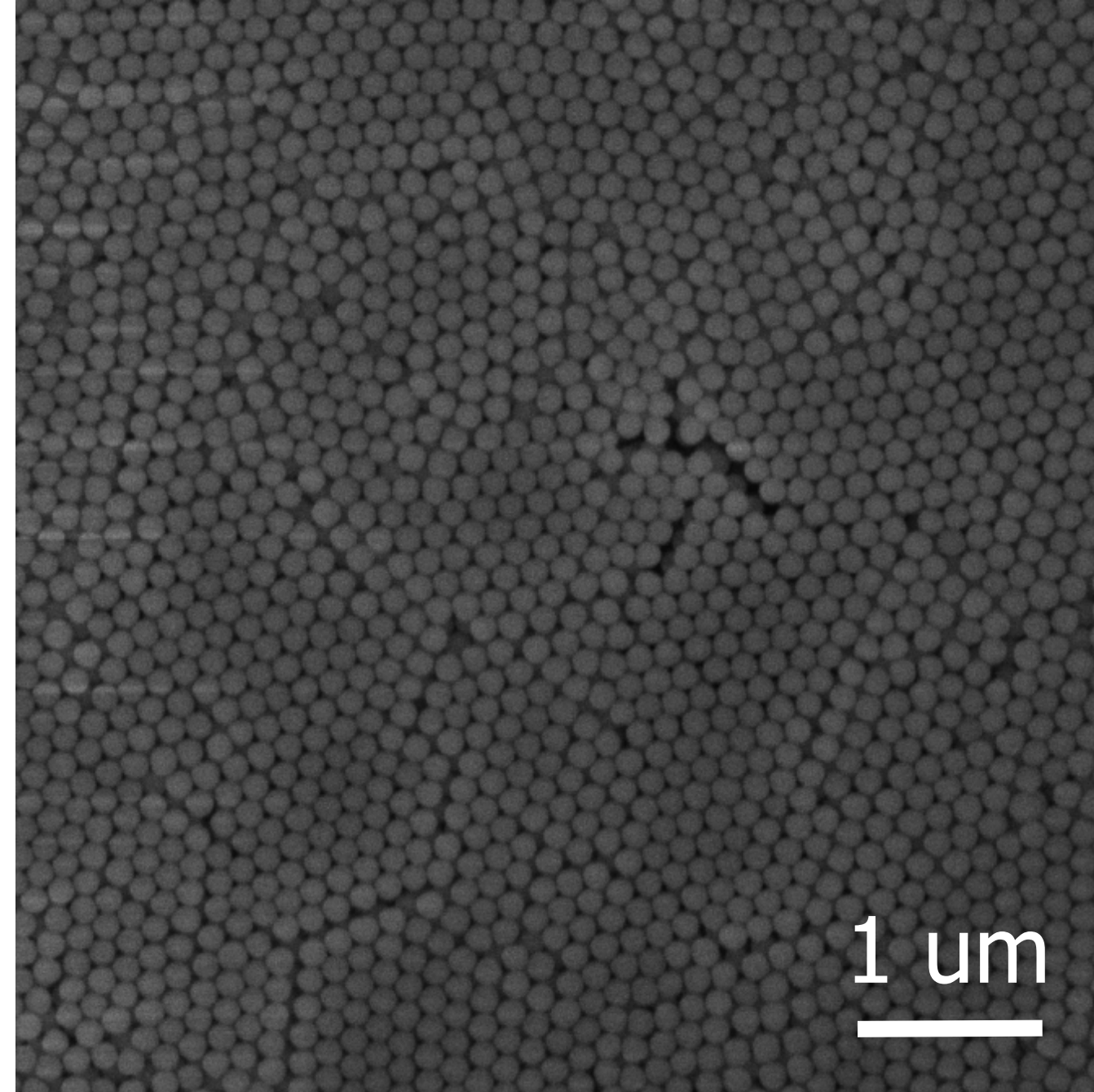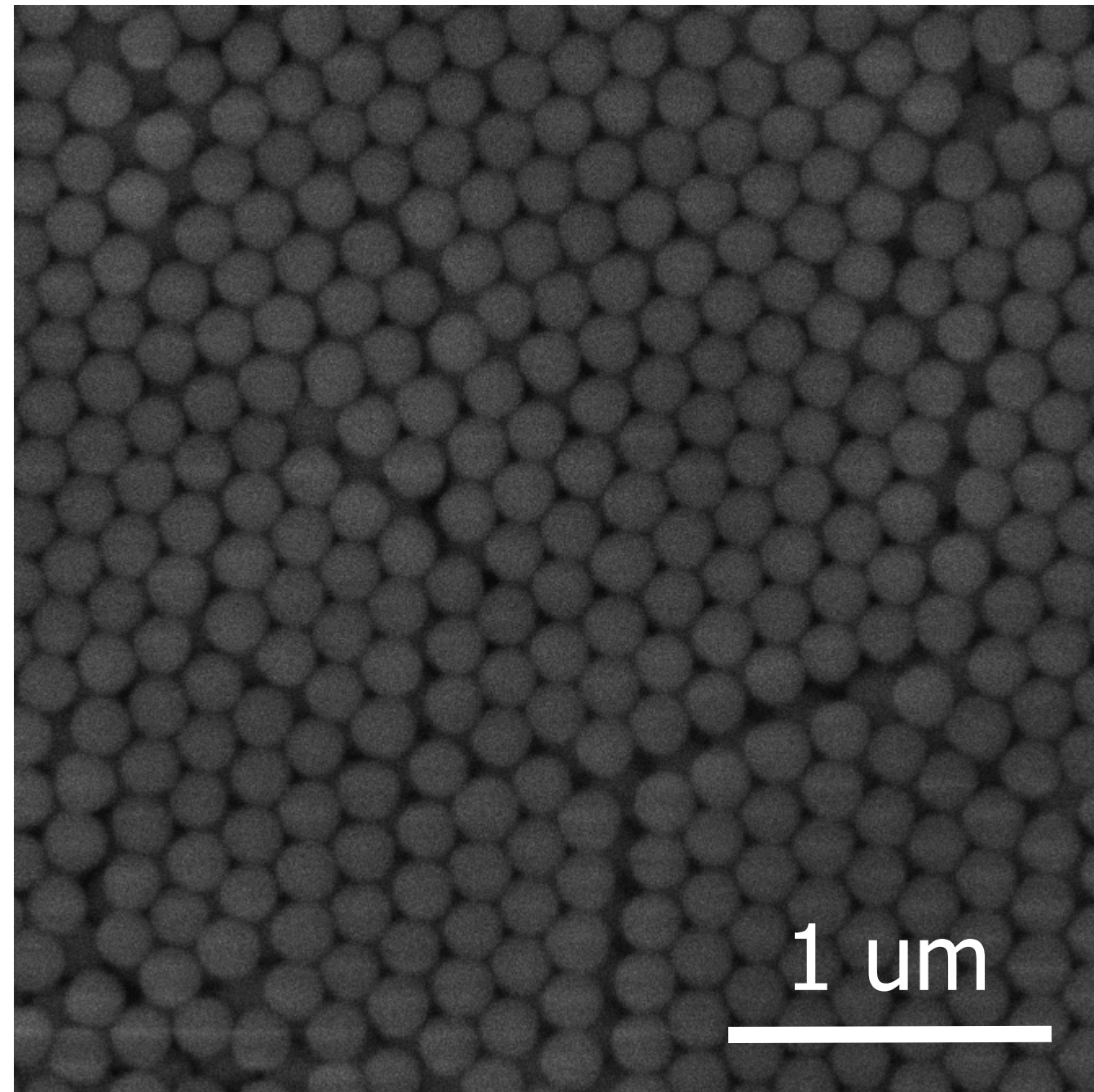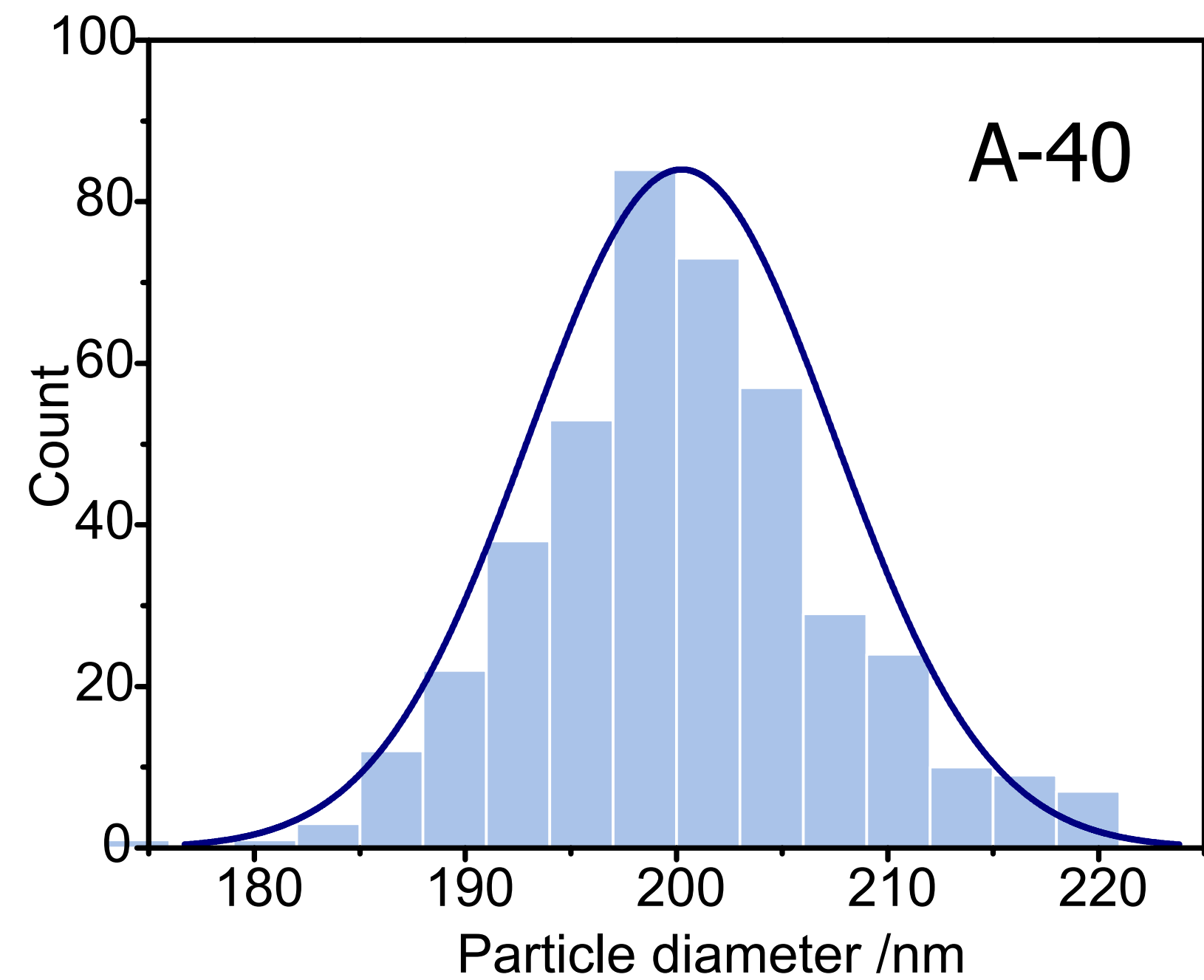

Supplement: Supplementary file 1 [file ijms-24-13693-s001.zip › Figures/SEM/200 nm.pdf]

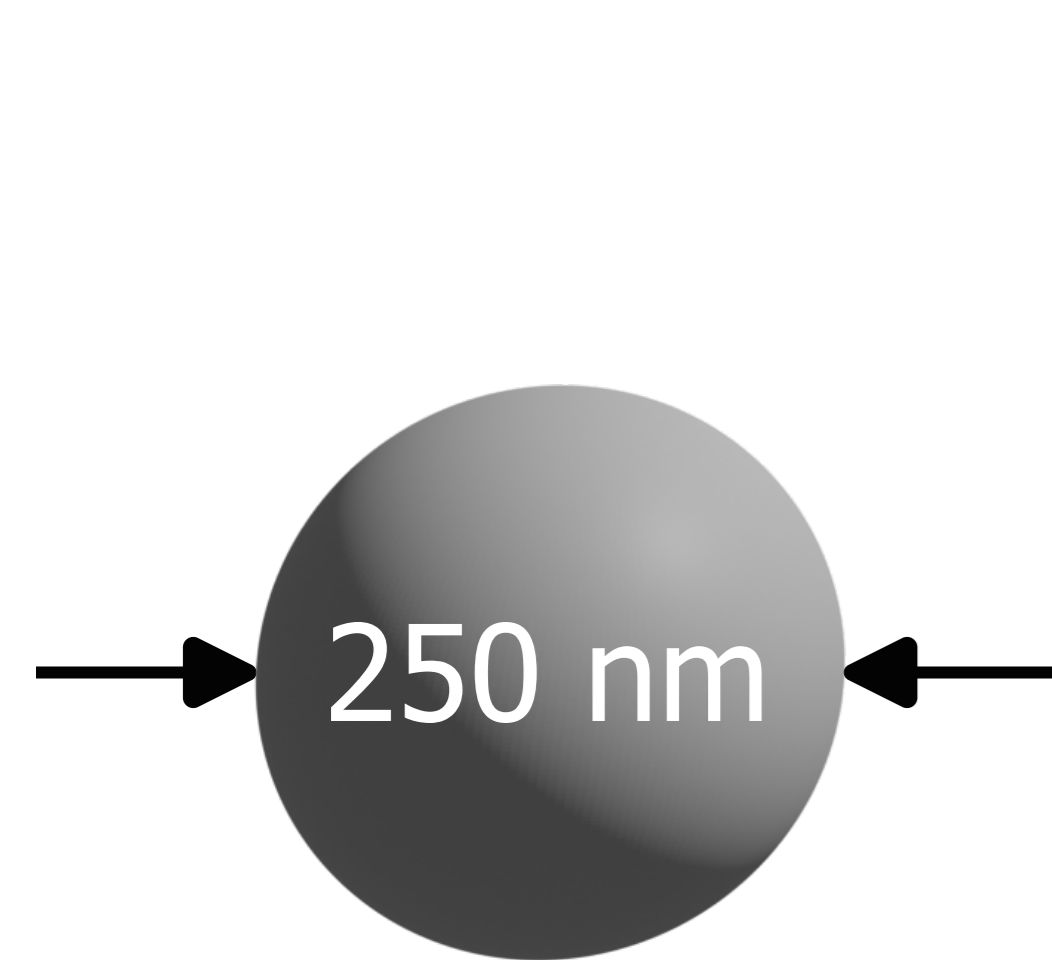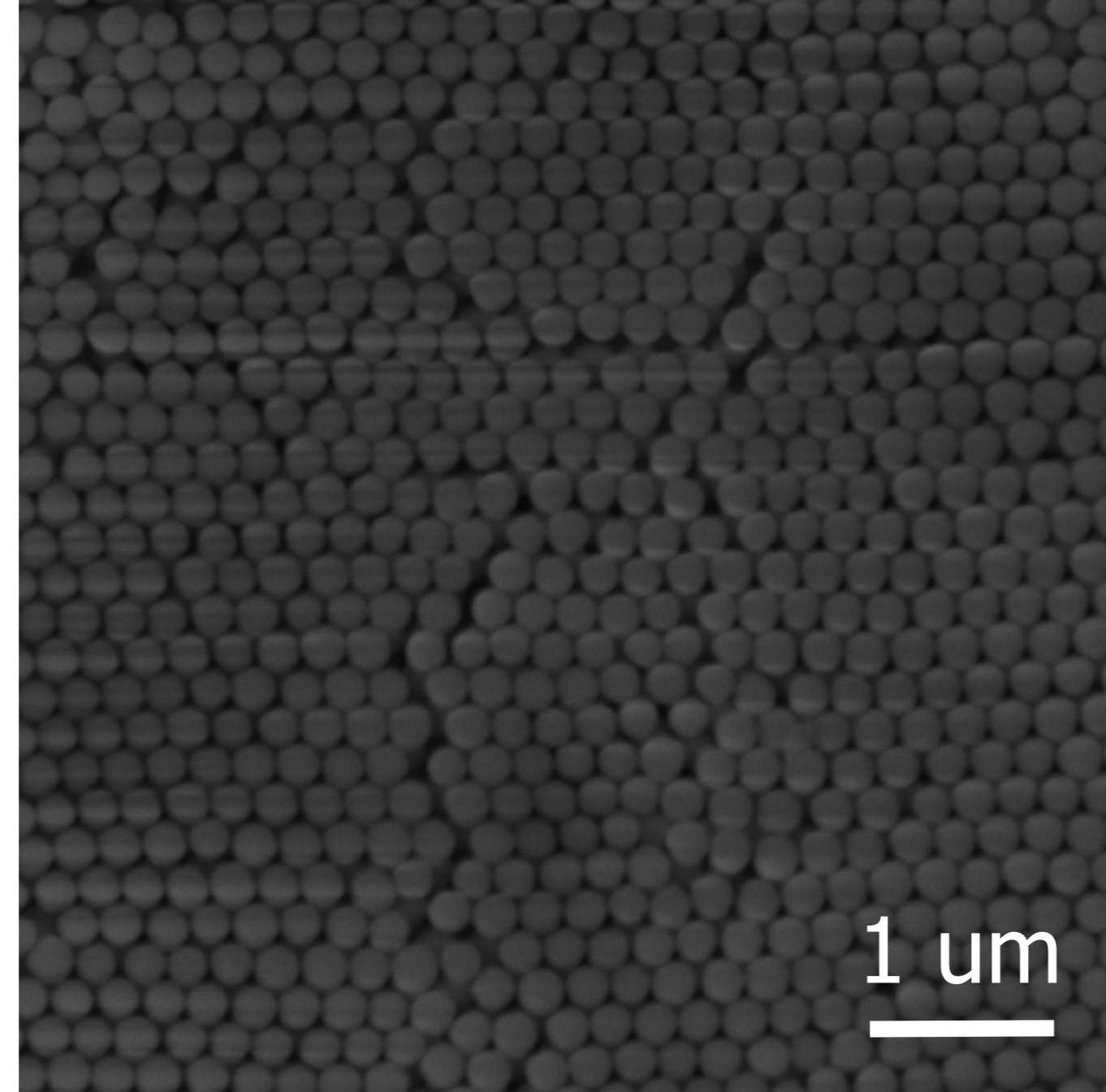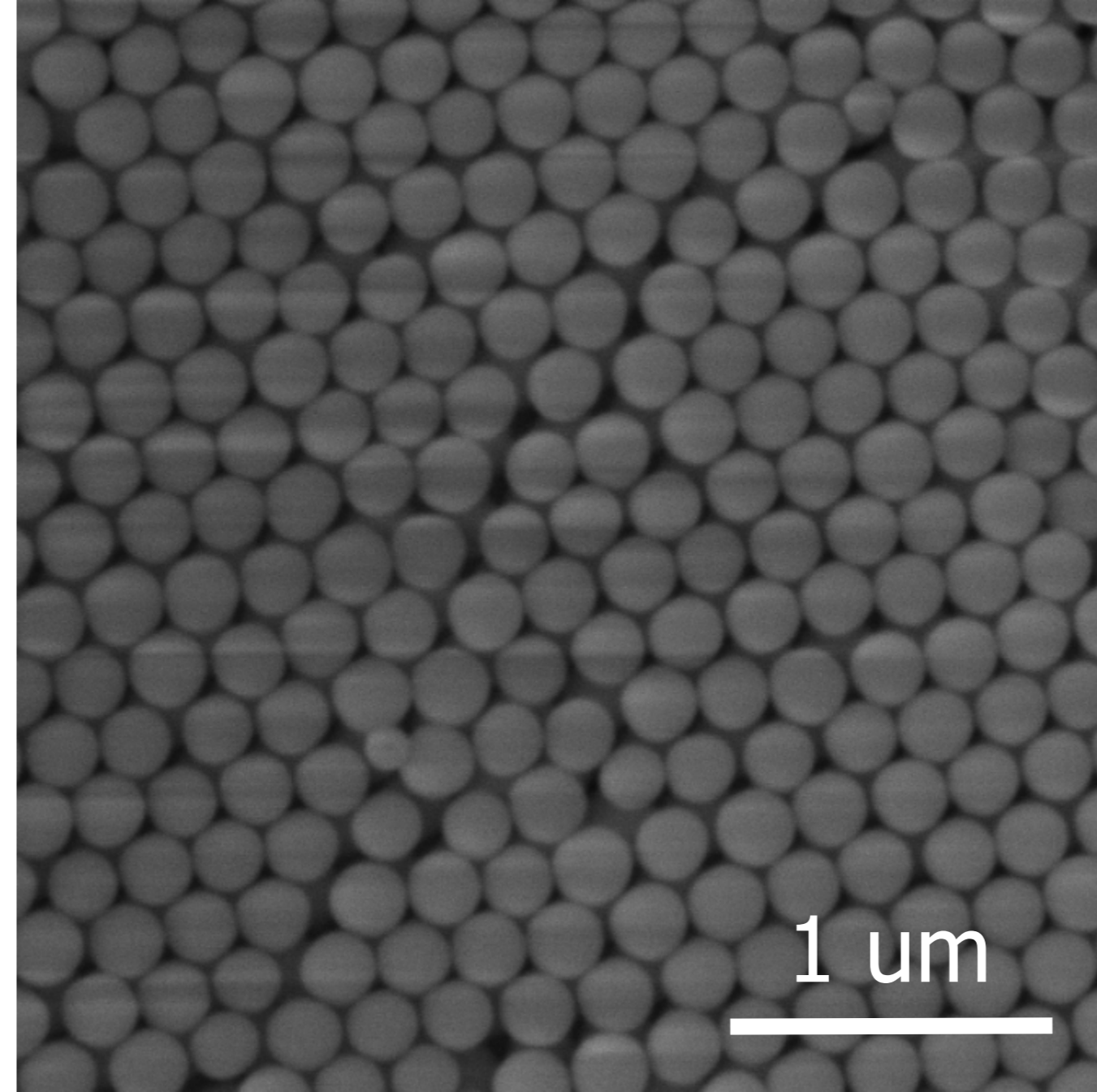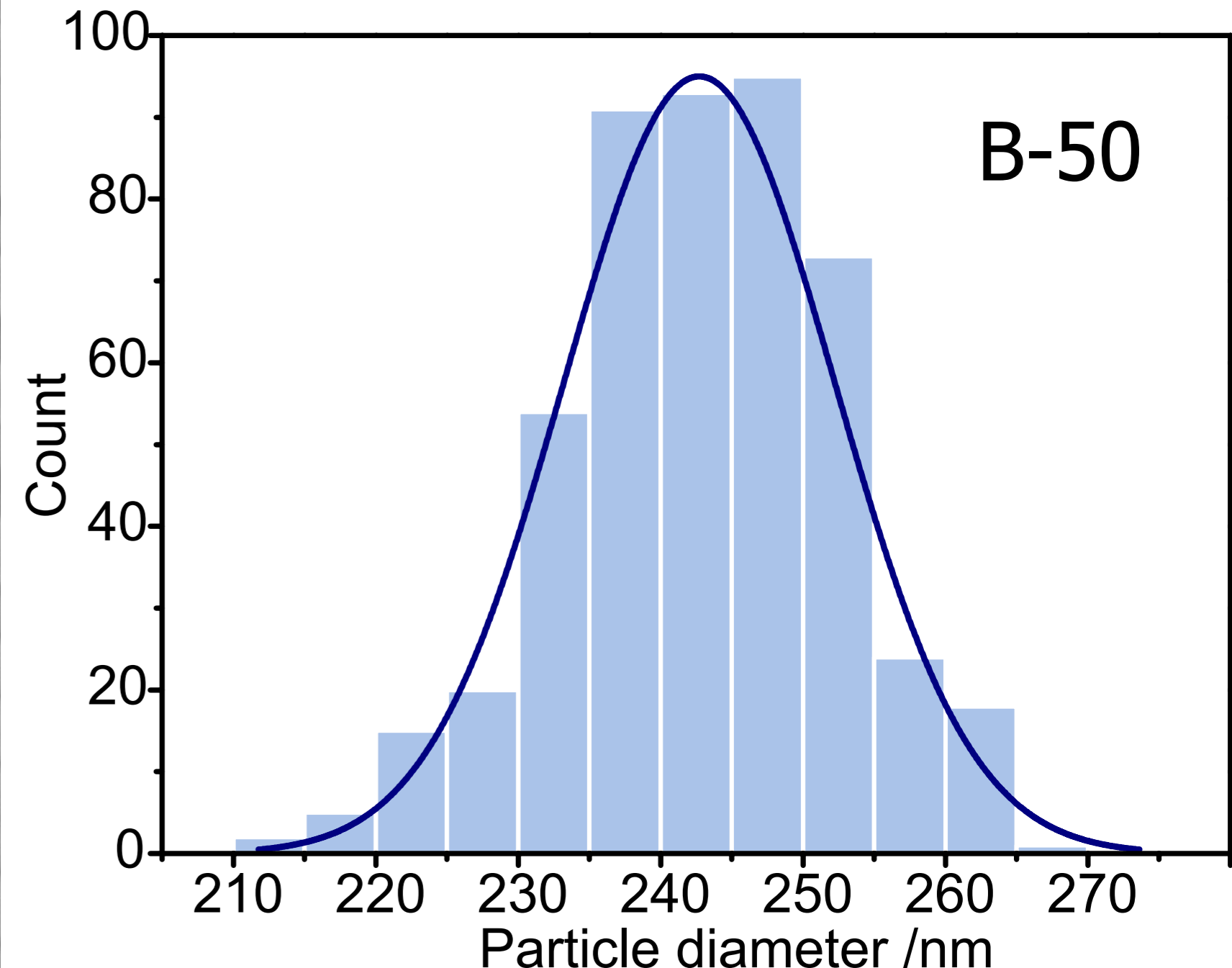

Supplement: Supplementary file 1 [file ijms-24-13693-s001.zip › Figures/SEM/250 nm.pdf]

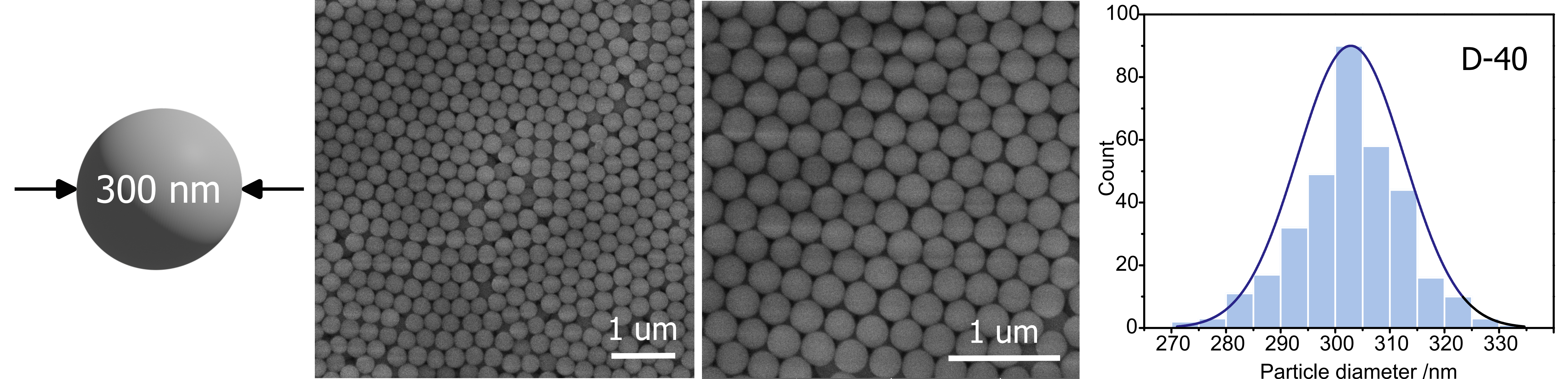

Supplement: Supplementary file 1 [file ijms-24-13693-s001.zip › Figures/SEM/300 nm.pdf]

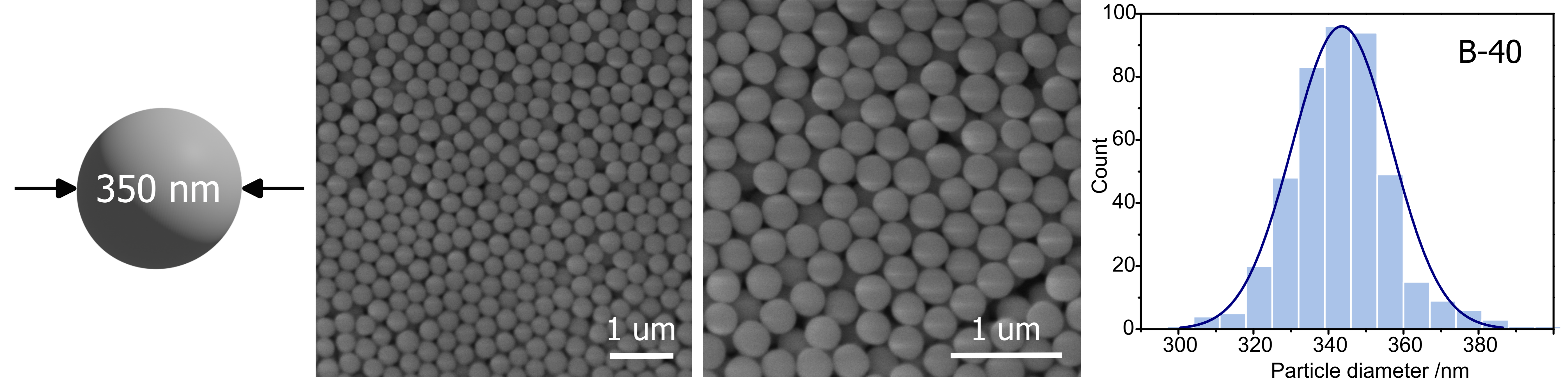

Supplement: Supplementary file 1 [file ijms-24-13693-s001.zip › Figures/SEM/350 nm.pdf]

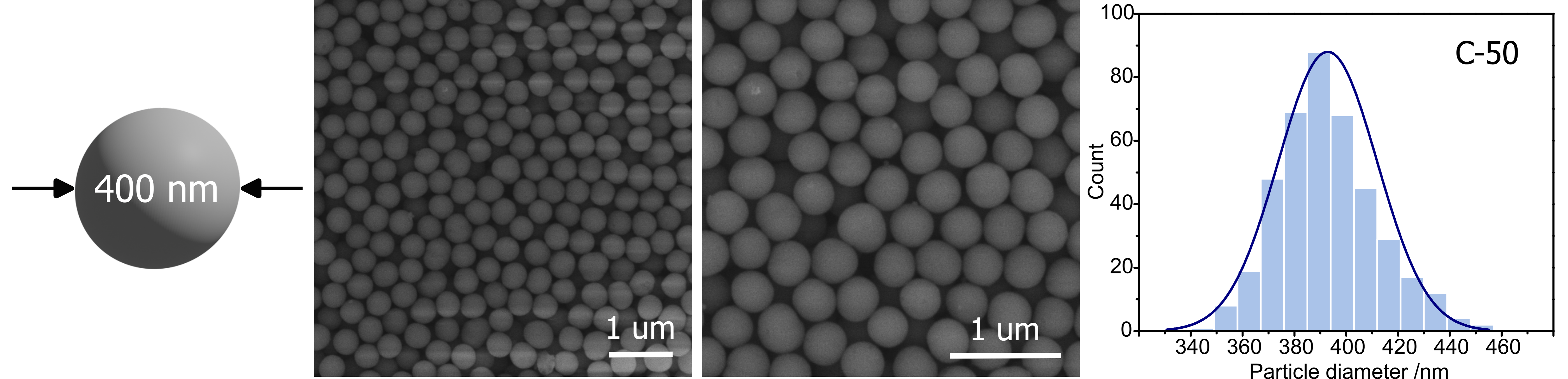

Supplement: Supplementary file 1 [file ijms-24-13693-s001.zip › Figures/SEM/400 nm.pdf]

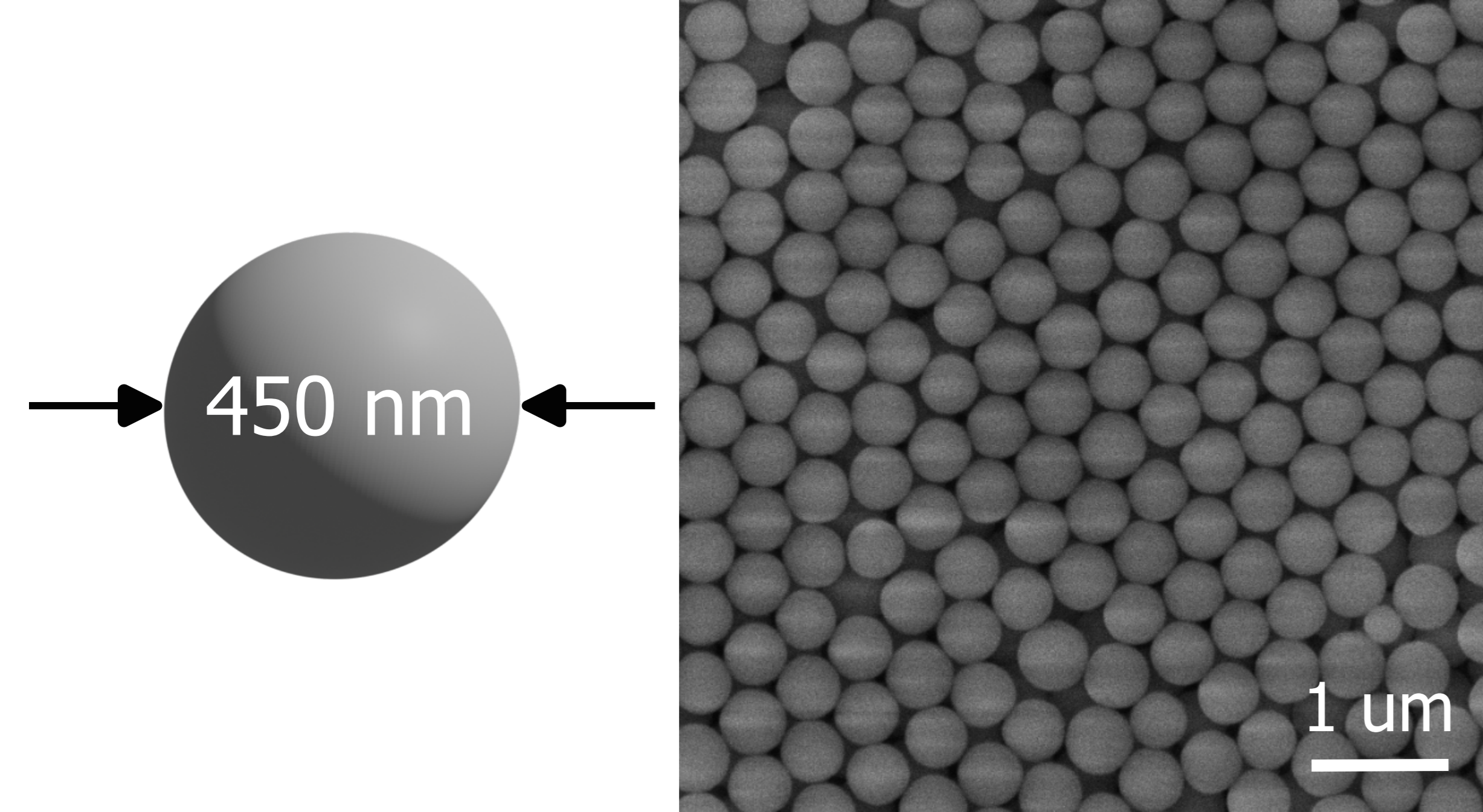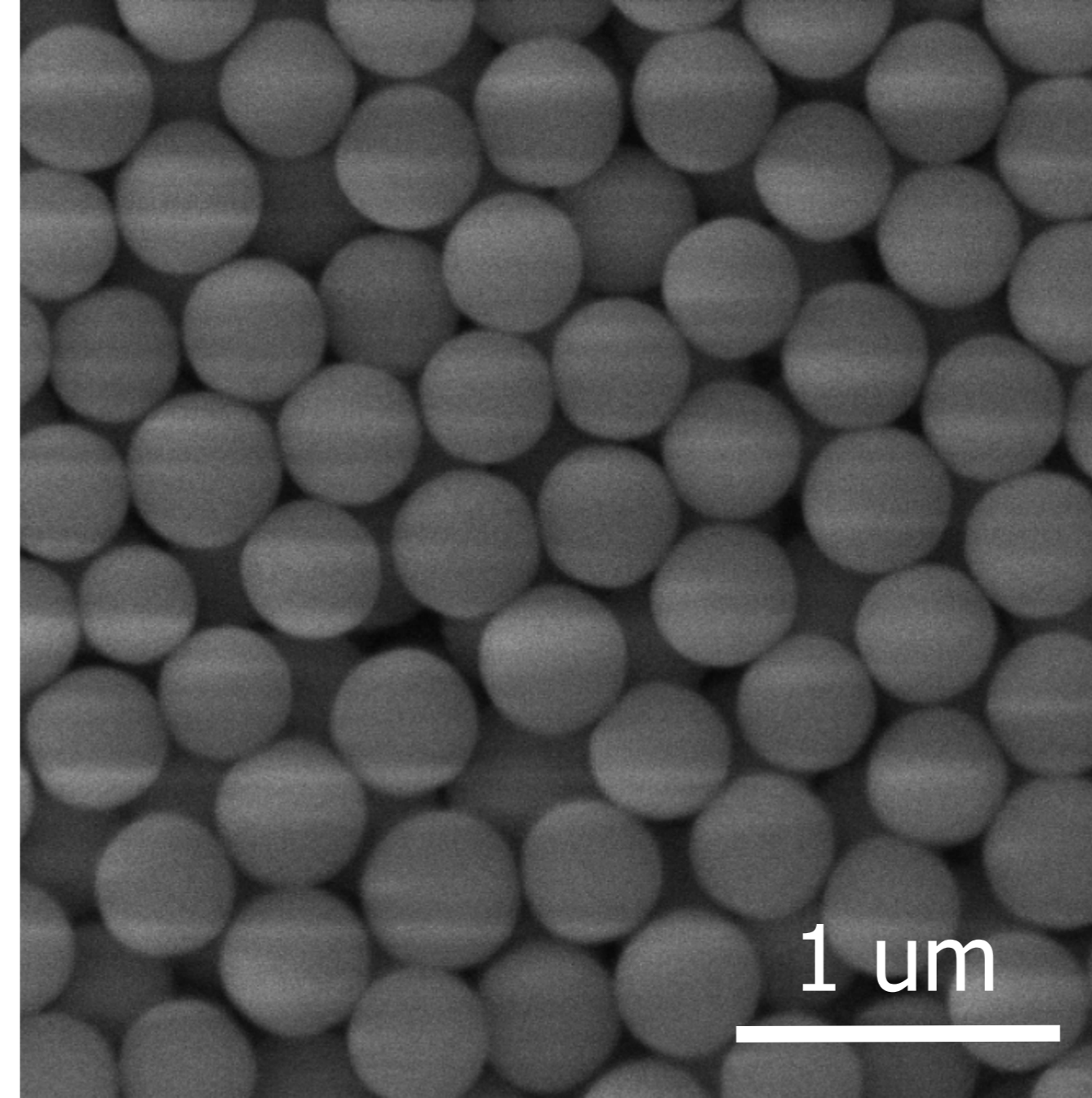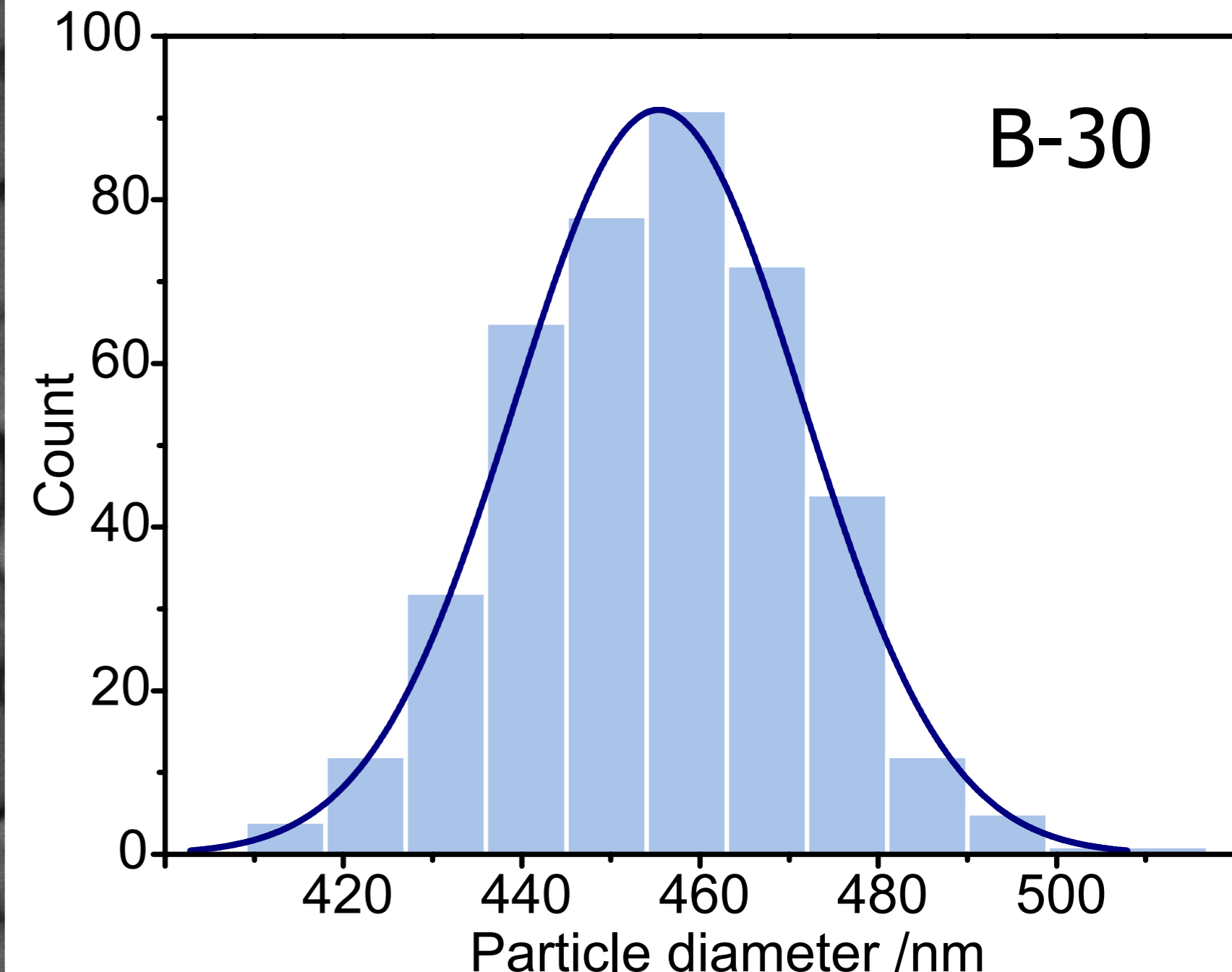

Supplement: Supplementary file 1 [file ijms-24-13693-s001.zip › Figures/SEM/450 nm.pdf]

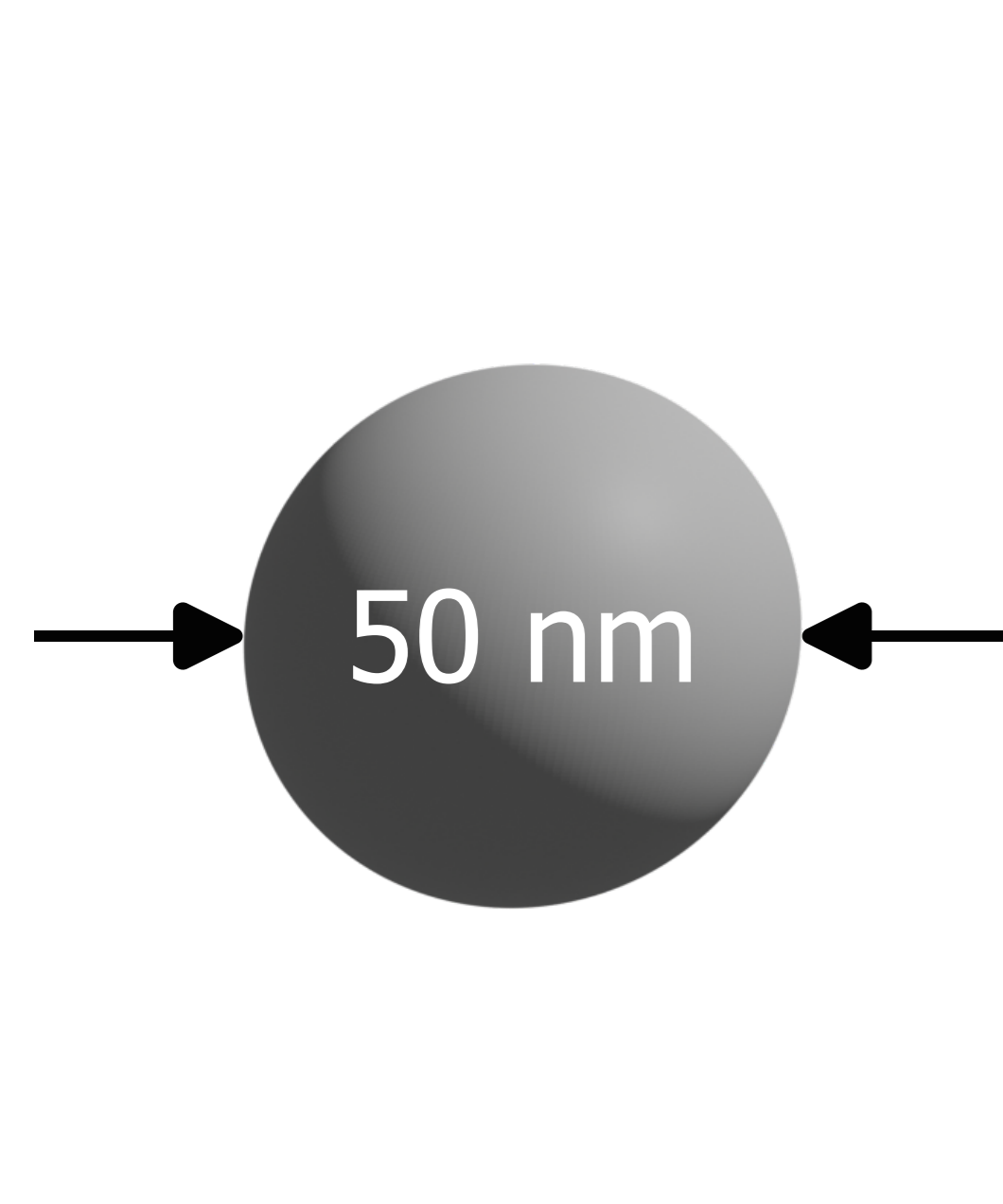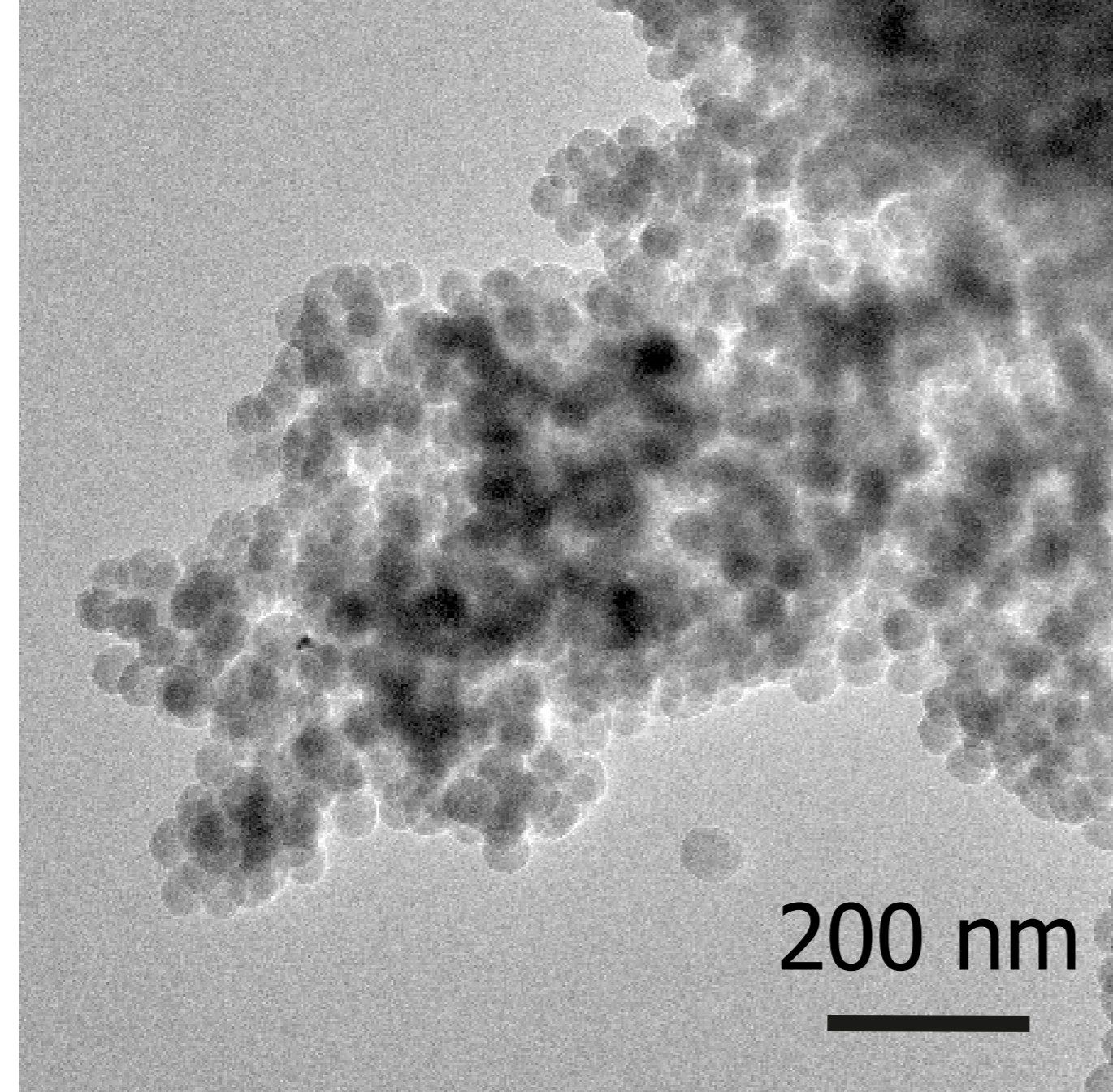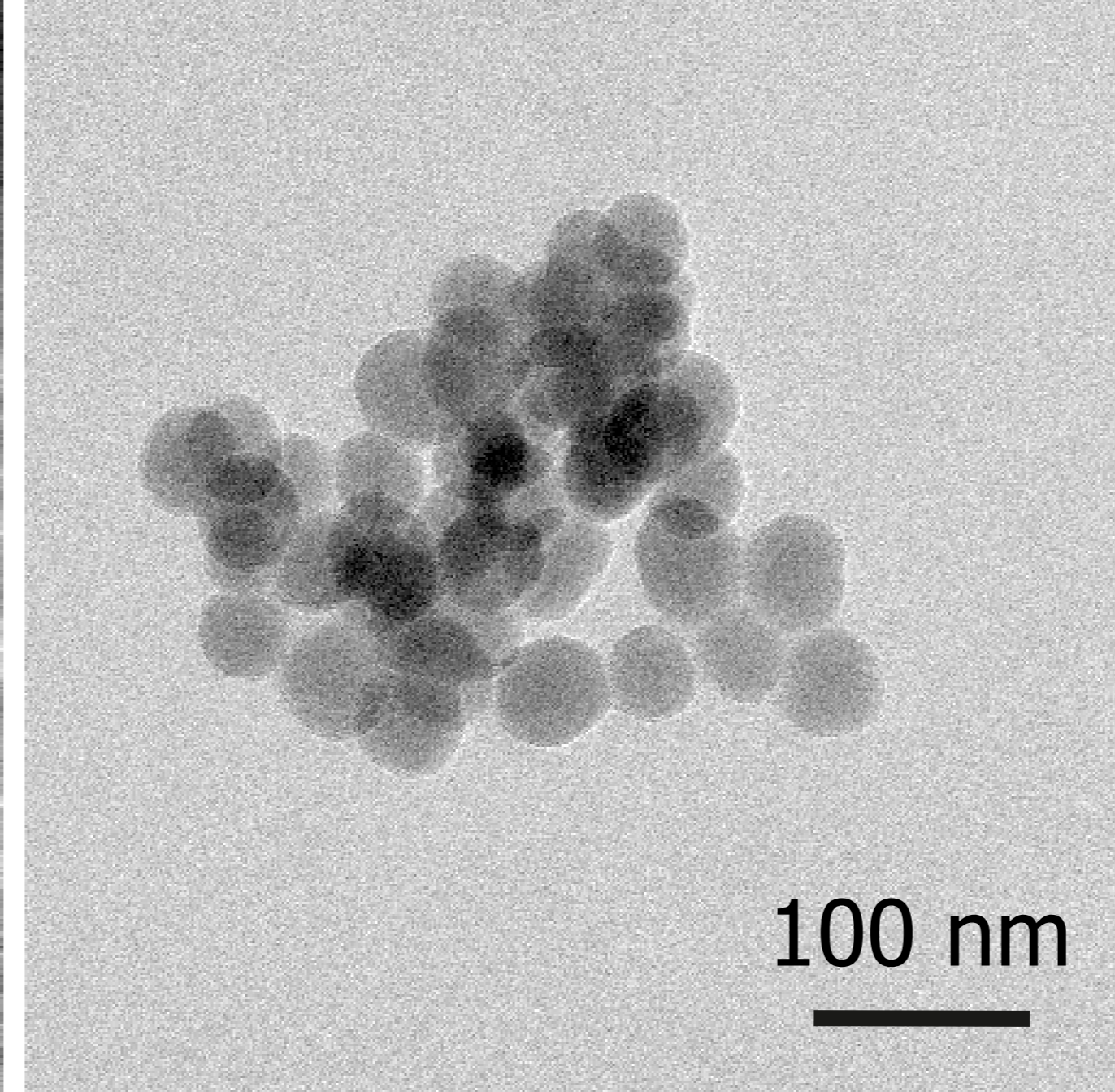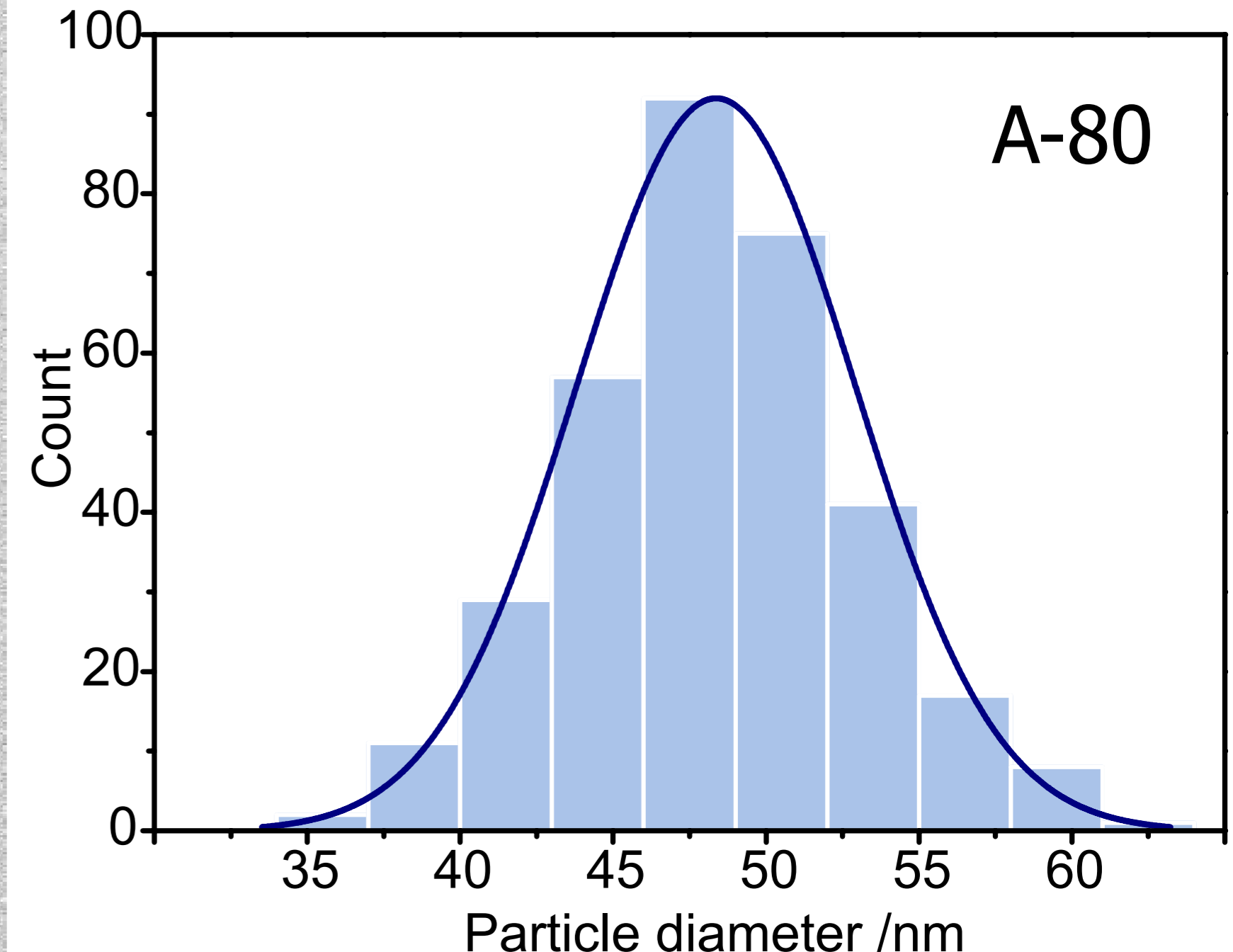

Supplement: Supplementary file 1 [file ijms-24-13693-s001.zip › Figures/SEM/50 nm.pdf]

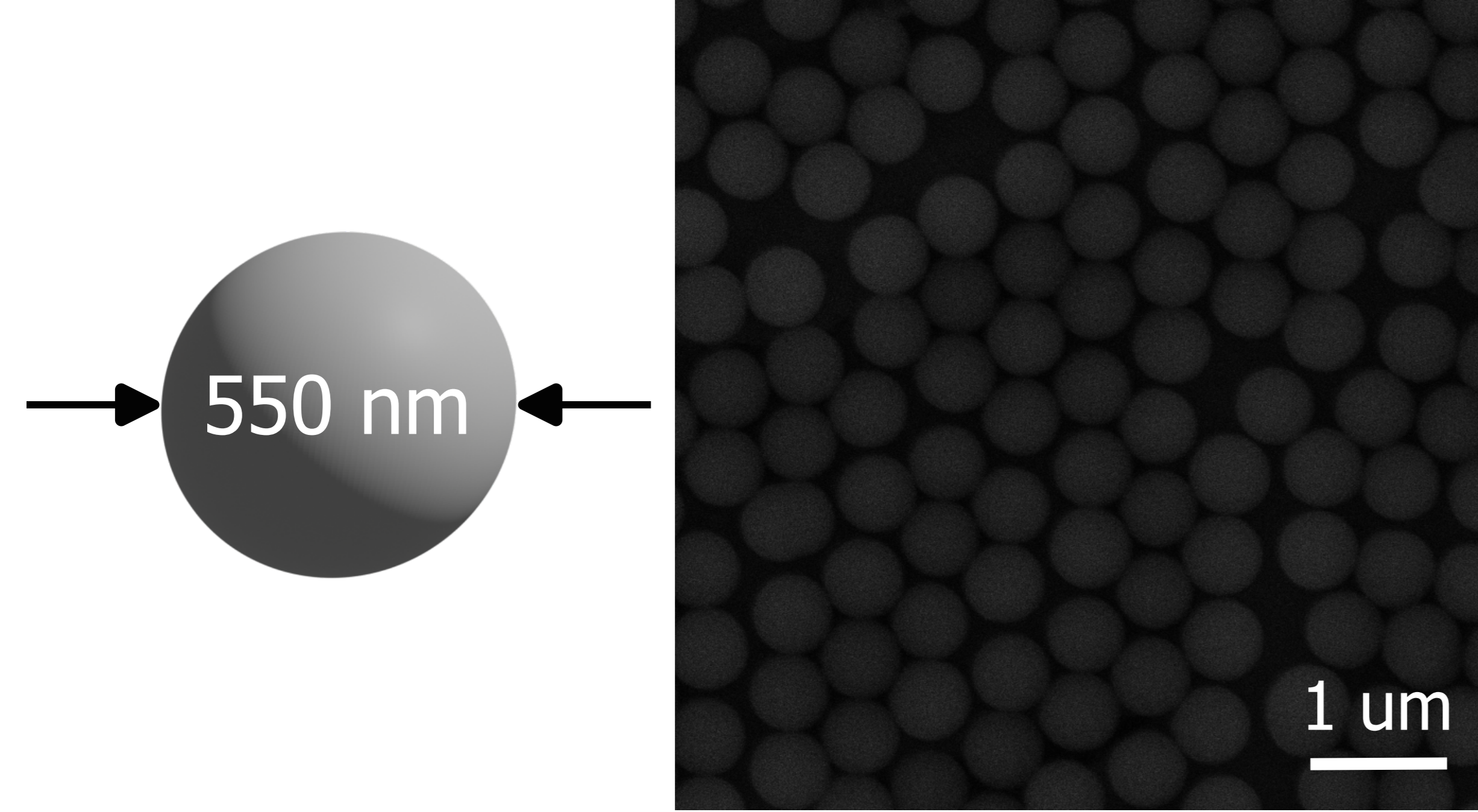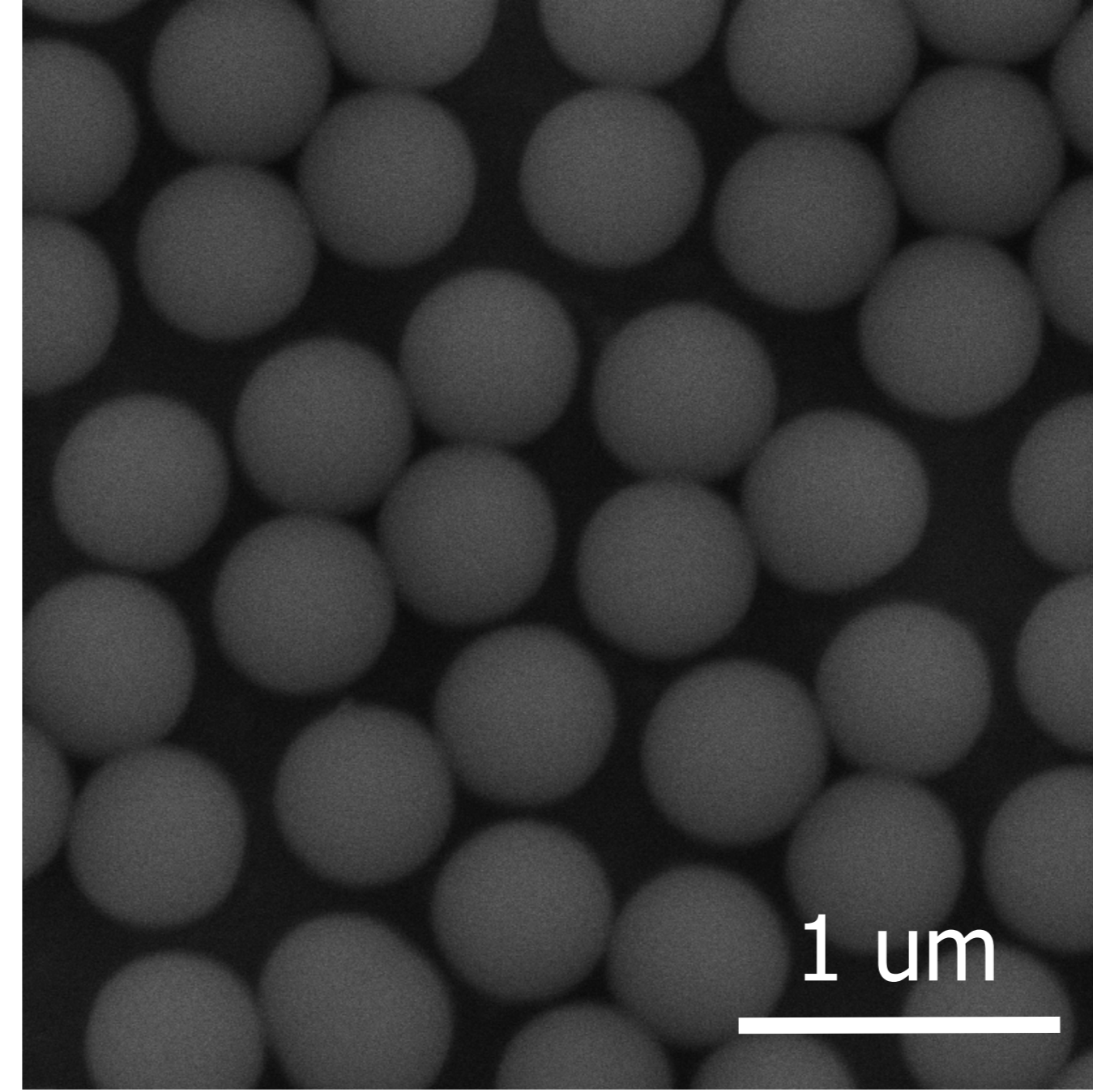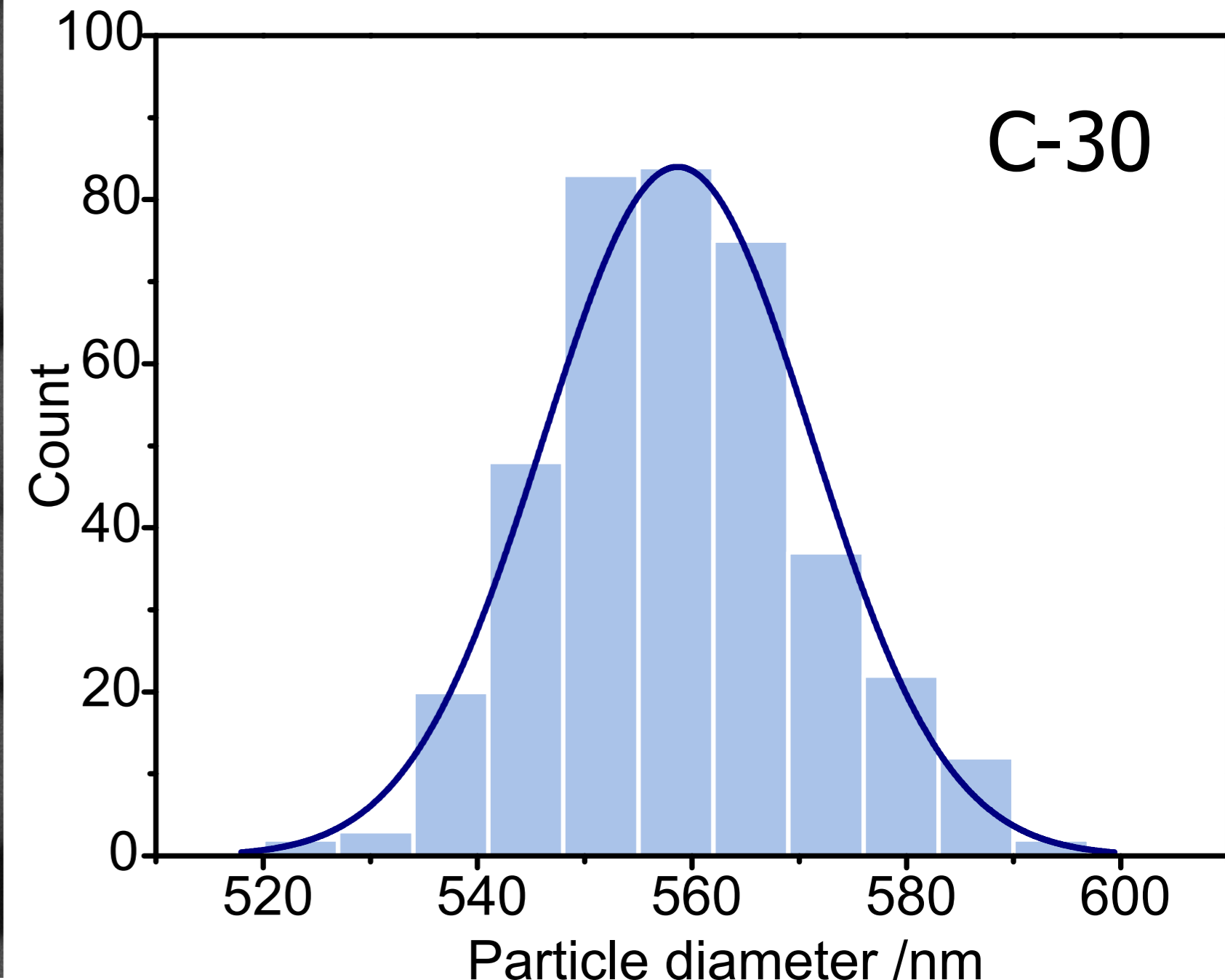

Supplement: Supplementary file 1 [file ijms-24-13693-s001.zip › Figures/SEM/550 nm.pdf]

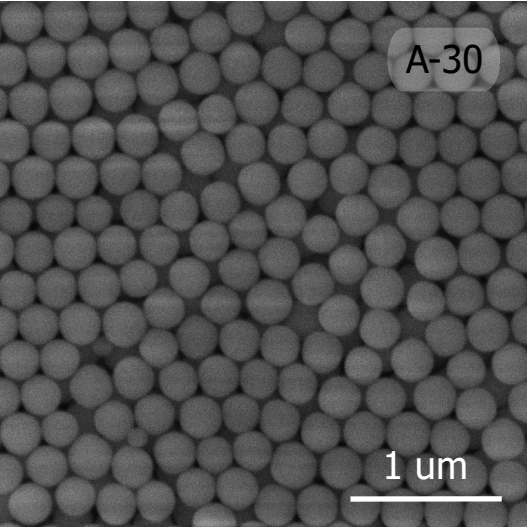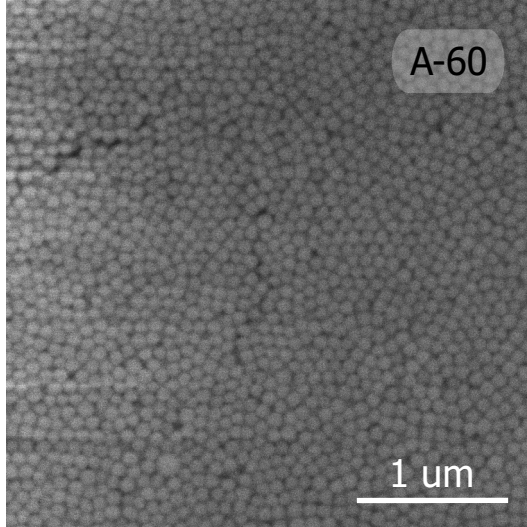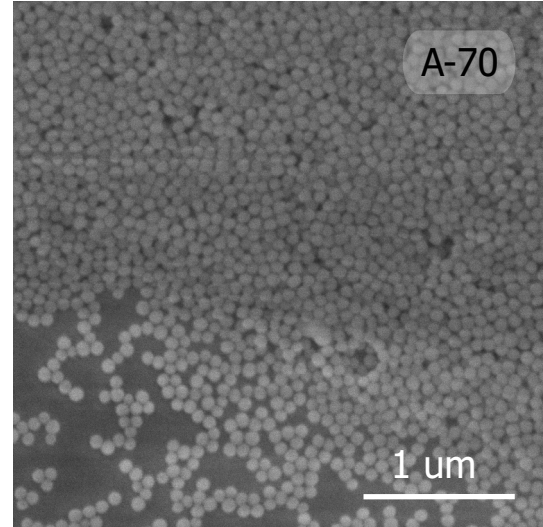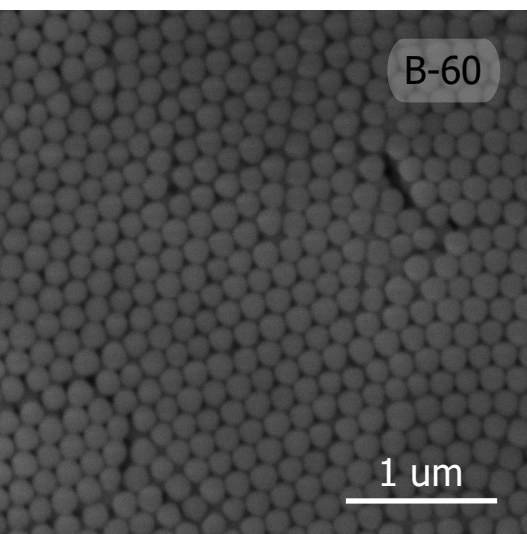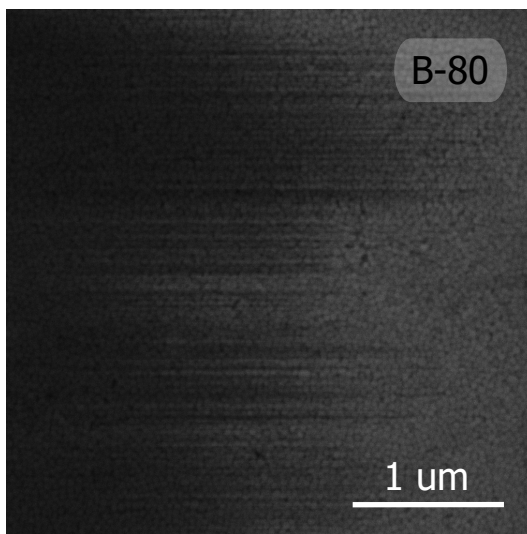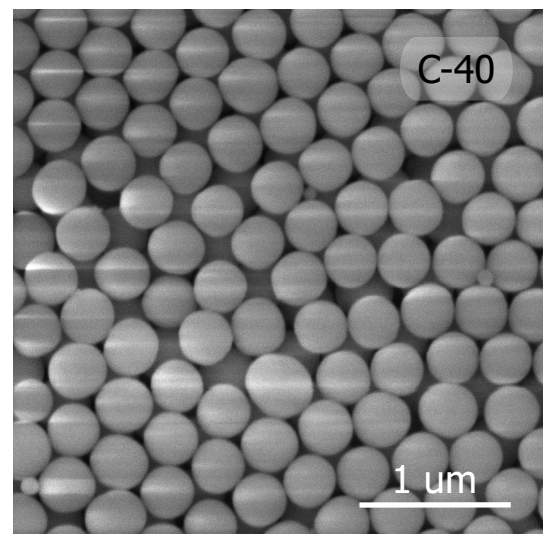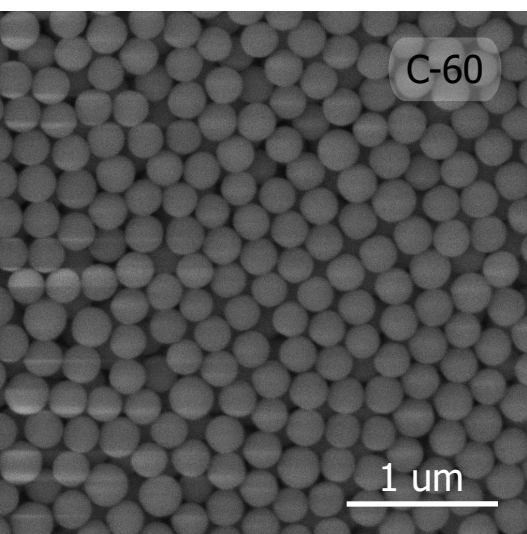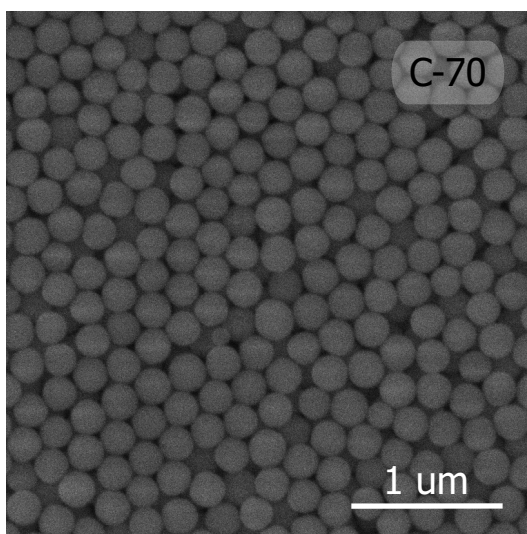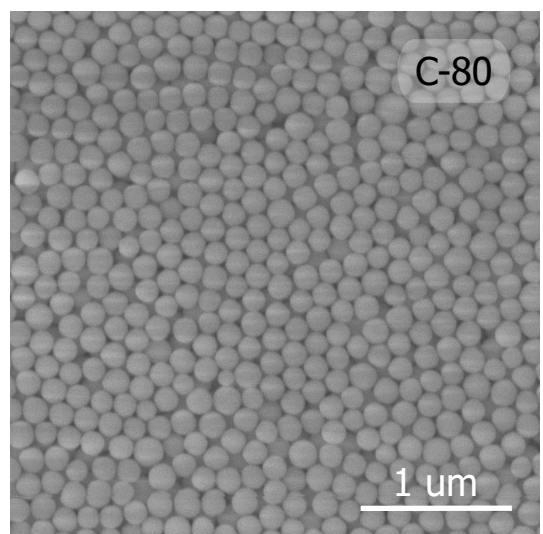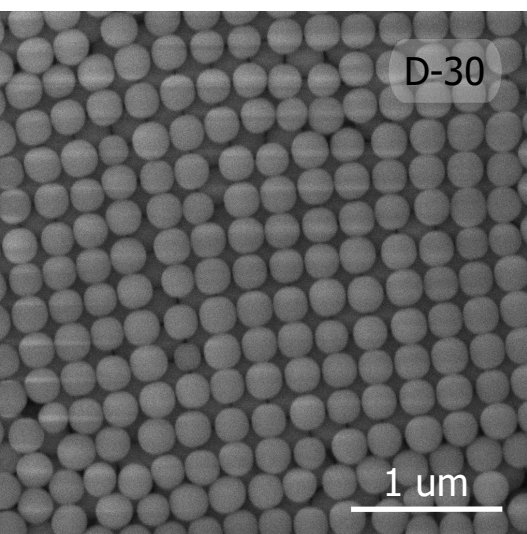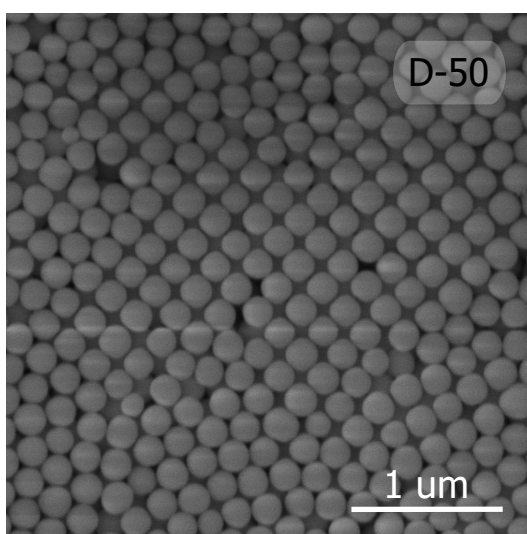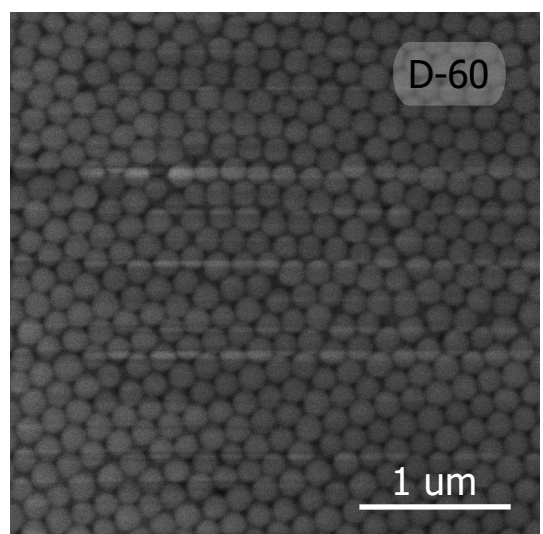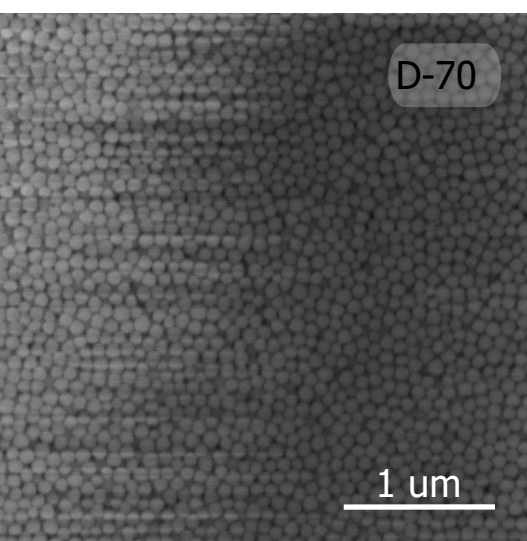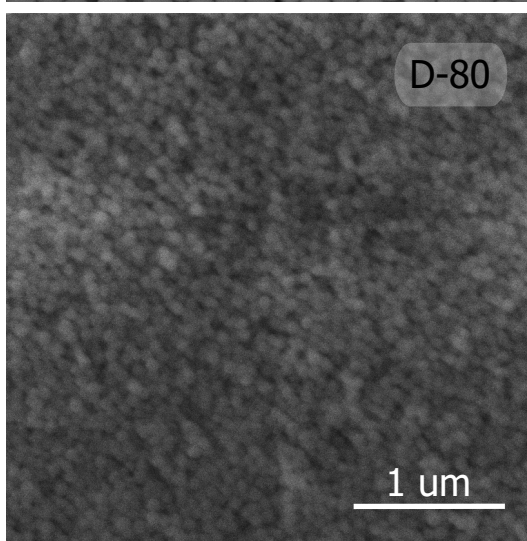

Supplement: Supplementary file 1 [file ijms-24-13693-s001.zip › Figures/SEM/SEM-SI.pdf]

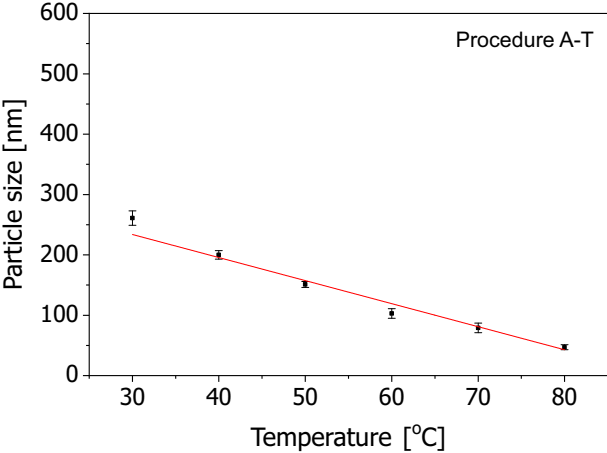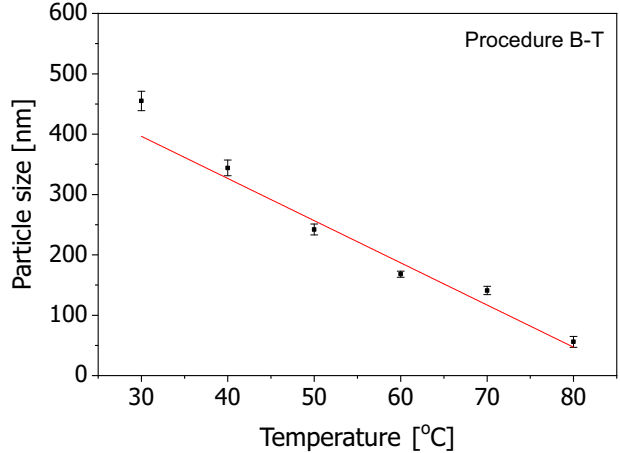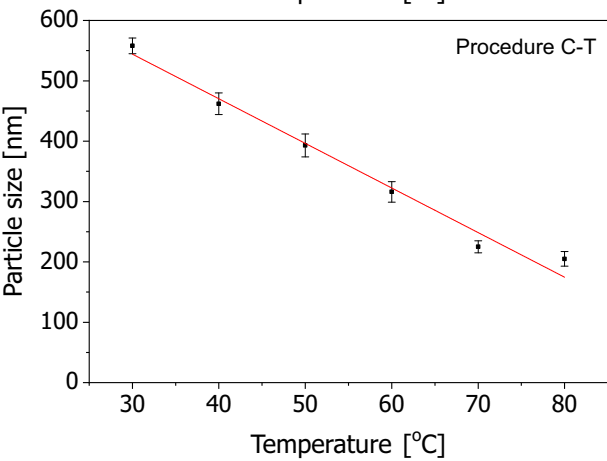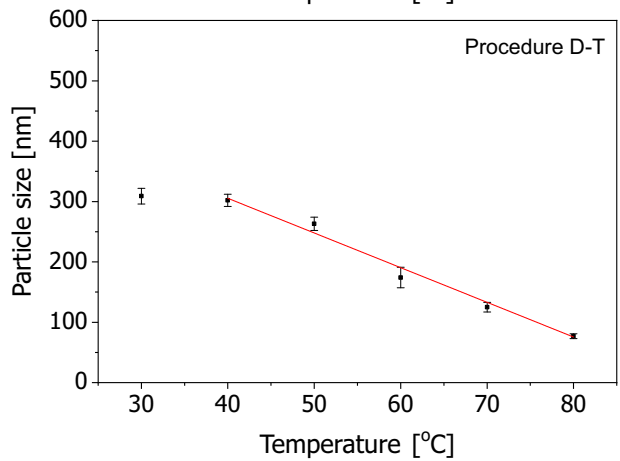

Supplement: Supplementary file 1 [file ijms-24-13693-s001.zip › Figures/SizesVsT.pdf]

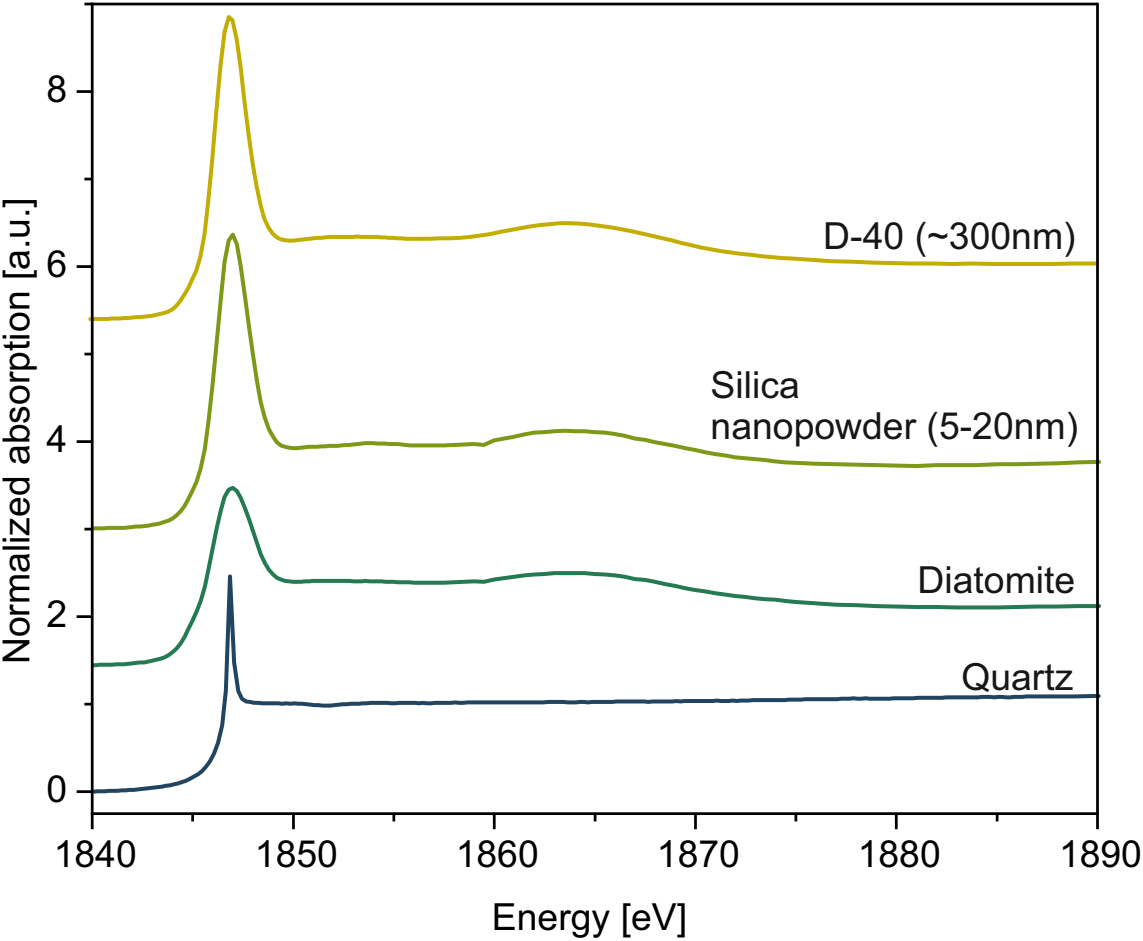

Supplement: Supplementary file 1 [file ijms-24-13693-s001.zip › Figures/XANES-silica-ref.pdf]

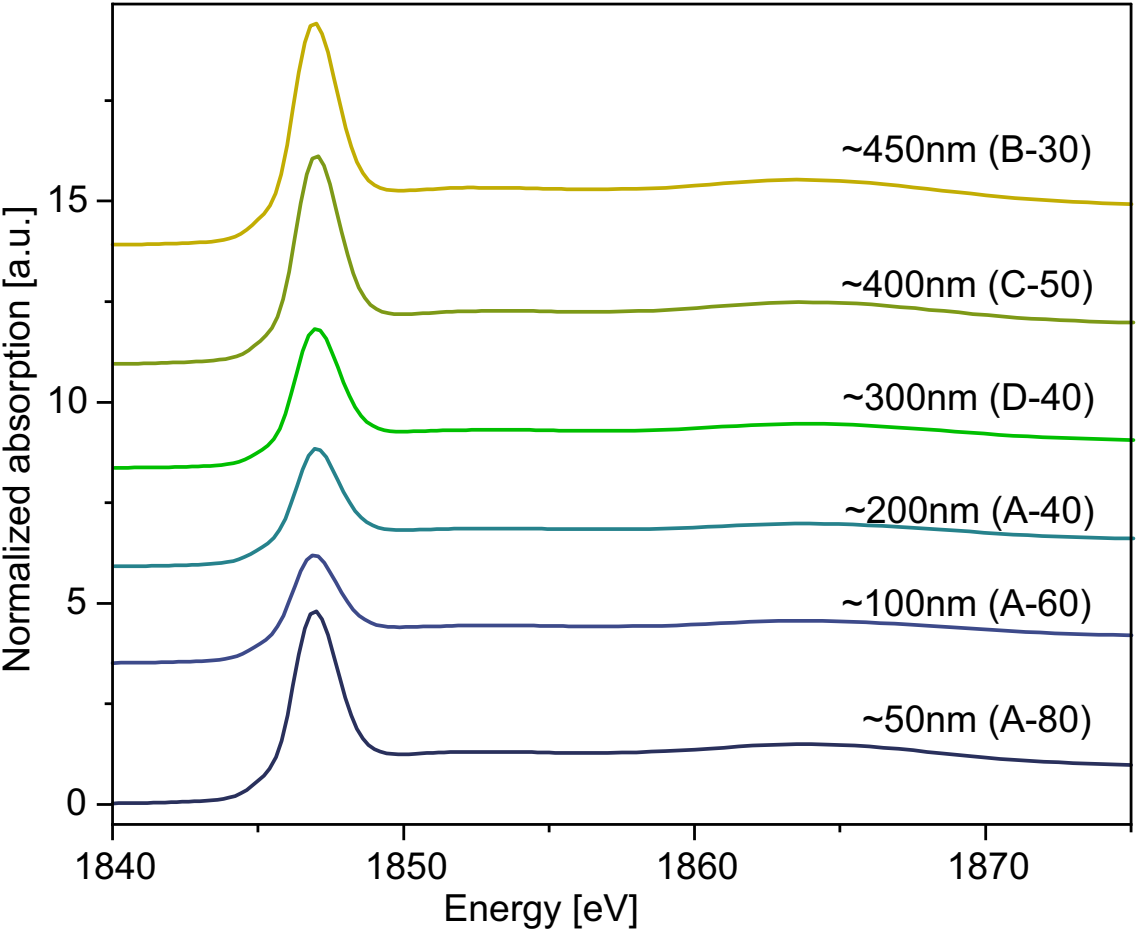

Supplement: Supplementary file 1 [file ijms-24-13693-s001.zip › Figures/XANES-silica.pdf]

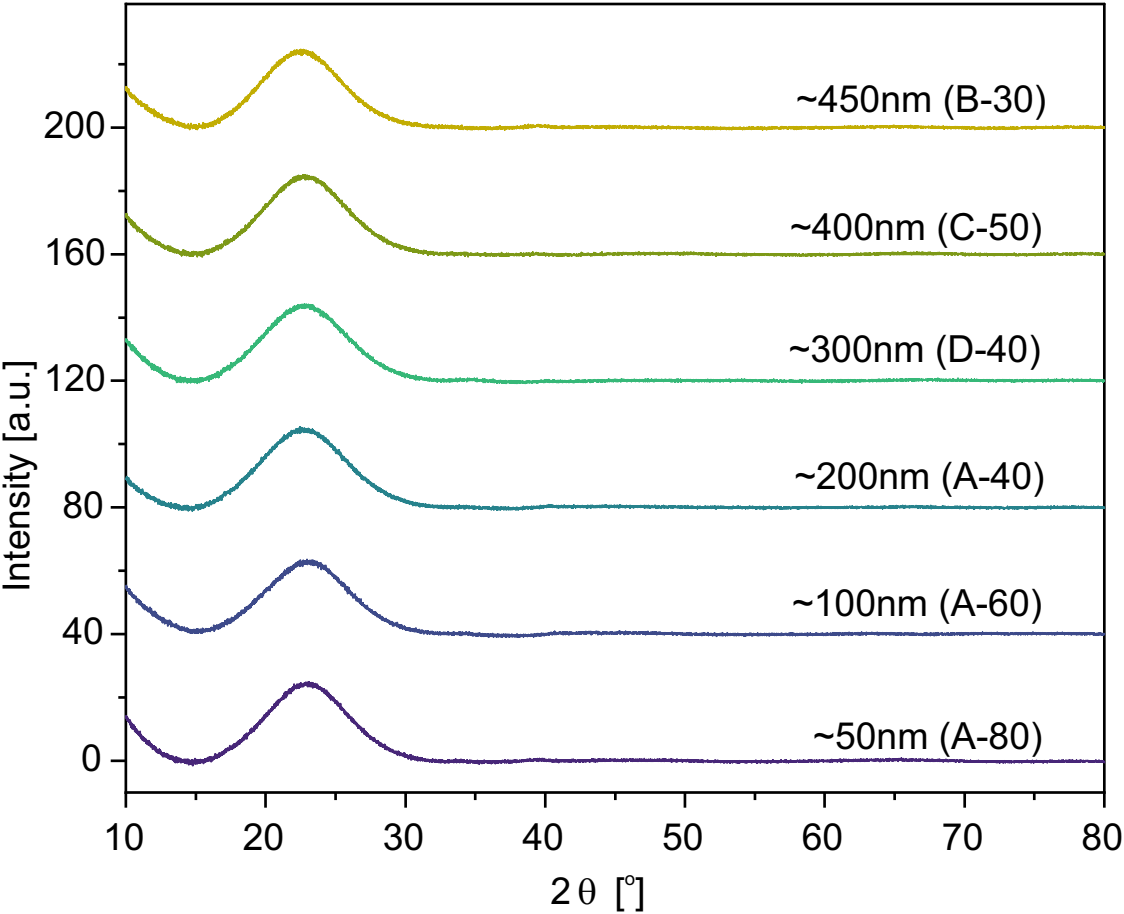

Supplement: Supplementary file 1 [file ijms-24-13693-s001.zip › Figures/XRD.pdf]
